# Contrast Gain Control in Auditory Cortex

**DOI:** 10.1016/j.neuron.2011.04.030

**Published:** 2011-06-23

**Authors:** Neil C. Rabinowitz, Ben D.B. Willmore, Jan W.H. Schnupp, Andrew J. King

**Affiliations:** 1 Department of Physiology, Anatomy, and Genetics, Sherrington Building, Parks Road, University of Oxford, Oxford OX1 3PT, UK

## Abstract

The auditory system must represent sounds with a wide range of statistical properties. One important property is the spectrotemporal contrast in the acoustic environment: the variation in sound pressure in each frequency band, relative to the mean pressure. We show that neurons in ferret auditory cortex rescale their gain to partially compensate for the spectrotemporal contrast of recent stimulation. When contrast is low, neurons increase their gain, becoming more sensitive to small changes in the stimulus, although the effectiveness of contrast gain control is reduced at low mean levels. Gain is primarily determined by contrast near each neuron's preferred frequency, but there is also a contribution from contrast in more distant frequency bands. Neural responses are modulated by contrast over timescales of ∼100 ms. By using contrast gain control to expand or compress the representation of its inputs, the auditory system may be seeking an efficient coding of natural sounds.

## Highlights

► We find evidence for spectrotemporal contrast gain control in auditory cortex ► Gain is determined by a combination of spectrally local and global contrast ► Within a limited range, mean stimulus level also affects neural gain ► Contrast gain control is fast (∼100 ms); gain decreases are faster than increases

## Introduction

The brain must be able to detect and represent both small and large changes in sound level. Not only do we experience a wide range of sound levels, from the quietness of a night in the forest to the hooting drama of crossing a street, but the important sensory information within these contexts may lie either in small or large deviations from the average sound. For example, detecting a subtle increase in the loudness of an approaching car's engine in a mostly constant background of traffic noise can be just as crucial as hearing a pronounced honk. This highlights a fundamental challenge for the auditory system: using neurons with limited dynamic range, the system has to represent large changes in sounds that are highly variable (high contrast), without losing the ability to represent subtle changes in sounds whose level is relatively constant (low contrast).

One way of managing a range of contrasts is to use separate circuits to process stimuli with different statistics. However, maintaining such a division-of-labor strategy across a sensory pathway requires a potentially costly duplication of resources. A more efficient solution is contrast gain control—where the responsiveness of neurons is dynamically adjusted according to the contrast of recent stimulation. Considerable evidence suggests that the mammalian visual system uses contrast gain control (Shapley and Victor, 1978) so that it can operate in both high- and low-contrast environments. This mechanism is well described by “divisive normalization,” whereby the range of visual input is adjusted according to the contrast of recent visual stimulation (Heeger, 1992; Carandini et al., 1997; Schwartz and Simoncelli, 2001; Bonin et al., 2005).

In the auditory system, several studies have investigated the effects of temporal (i.e., within-band) contrast on neural responses and have provided evidence both for gain control and for multiple independent circuits. A simple way of controlling temporal contrast is to vary the modulation depth of sinusoidally amplitude-modulated tones; neurons from the auditory nerve (Joris and Yin, 1992) to the auditory cortex (Malone et al., 2007) can rescale their gain to partially compensate for reduced modulation depths. Similar effects have been found in the inferior colliculus (IC) (Kvale and Schreiner, 2004; Dean et al., 2005) and in the songbird forebrain (Nagel and Doupe, 2006) when the temporal contrast of more complex stimuli is altered. Such gain changes improve the efficiency with which neurons encode frequently presented levels (Dean et al., 2005).

Other studies have found that mean firing rates of IC neurons can have nonmonotonic dependencies on spectrotemporal contrast, while retaining their spectrotemporal preferences (Escabí et al., 2003). Similar tuning of mean firing rate to spectral contrast (measured across frequency, but not across time) has been reported in auditory cortex (Barbour and Wang, 2003). These findings suggest a division-of-labor strategy. However, such effects are also compatible with contrast gain control, so long as gain changes are slow (compared to spike generation) or do not completely compensate for changes in contrast.

In this study, we ask whether the mammalian auditory cortex adjusts neural gain according to the spectrotemporal contrast of recent stimulation. One possibility is that neurons' responses are invariant to the statistics of recent stimulation, suggesting that the problem is ignored. Alternatively, neurons may be informative only about stimuli with a particular contrast, suggesting a division-of-labor strategy. Finally, they may undergo more complex changes in their spectrotemporal tuning as contrast varies, suggesting a reallocation of resources in the auditory system. Tuning of auditory cortical neurons has been shown to depend on stimulus context, such as tone density (Blake and Merzenich, 2002), stimulus bandwidth (Gourévitch et al., 2009), and the history of recent stimulation (Ahrens et al., 2008). To distinguish between these hypotheses, we designed a set of stimuli where the statistics of level variations could be controlled within individual frequency bands. This allowed us to measure the spiking responses of neurons in the auditory cortex to sounds with different means and contrasts, from which we estimated spectrotemporal receptive fields (STRFs), using both linear (deCharms et al., 1998; Schnupp et al., 2001) and linear-nonlinear (LN) (Chichilnisky, 2001; Simoncelli et al., 2004; Dahmen et al., 2010) models.

We also sought to quantify which combination of stimulus statistics might inform cortical gain control. This requires a formal definition of the contrast of a sound. In the visual system, the contrast of a simple stimulus is defined as the ratio of the intensity difference to the mean intensity (c=ΔI/I); this definition can be generalized to complex stimuli as the ratio of the standard deviation to the mean ($c=\sigma_{I}/\mu_{I}$). In principle, the same definitions can be applied directly in the auditory system. However, it is normal to describe sounds using sound pressure level (SPL), $L=20\;{\text{log}}_{10}\left(p/p_{REF}\right)$, rather than (RMS) pressure, *p*, itself. The effect of this log transform is that the standard deviation of the SPL of a sound (*σ_L_*) can provide an excellent approximation of the contrast, *σ_P_*/*μ_P_*, of the sound pressure:

(1) $$c=\frac{\sigma_{P}}{\mu_{P}}\approx a.\sigma_{L}+b$$

Thus, an auditory contrast gain mechanism would adjust neural gain according to *σ_L_*, the standard deviation of the SPL of recent stimulation.

Finally, we investigated whether gain control is a local or a network mechanism. If a neuron's gain depends only on the statistics of the stimuli presented within its STRF, then gain control could be implemented locally, e.g., by synaptic depression within individual neurons (Carandini et al., 2002). However, synaptic depression is unlikely to account for gain effects that result from the statistics of stimuli outside the STRF, in which case gain control is more likely to arise from network mechanisms, such as the leveraging of balanced excitation and inhibition (e.g., Mante et al., 2005). We therefore changed the stimulus contrast both inside and outside narrow frequency bands in our stimuli, in order to assess whether neuronal sensitivity to small changes in a sound depends on the statistics of its spectrally local or more global context.

## Results

**Figure 1:**
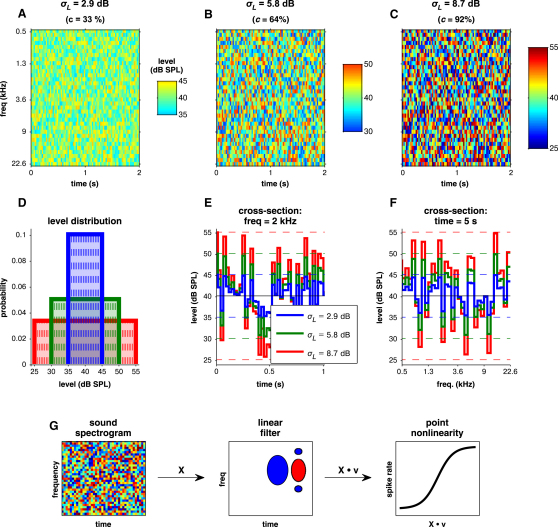
Stimulus Paradigm (A–C) Dynamic random chord (DRC) sequences with different spectrotemporal contrasts. The elements of each sequence are chords of pure tones, whose levels are randomly chosen from the distributions in (D). (D) Tone level distributions for the DRC sequences in (A)–(C). These all have the same mean level *μ_L_*, but different widths. The blue line corresponds to the low-contrast (*σ_L_*) DRC (A); the green line is the medium-contrast DRC (B); the red is the high-contrast DRC (C). (E and F) The widths of the level distribution determine both the spectral and the temporal contrast of the individual sequences, as shown by the temporal profiles of a pure tone at a fixed frequency for the three sequences in (E) and the spectral profiles of a chord at a fixed time for the three sequences in (F). (G) We characterized the relationship between stimulus and neuronal response using a linear-nonlinear model. The sound input is treated as a spectrogram, **X**; the (normalized) receptive field **v** acts as a linear filter on **X**, extracting the relevant features of the sound via the dot product X⋅v. The output of the linear filter is (optionally) matched to the output spike rate through a nonlinearity that captures features such as thresholding. We parameterized these nonlinearities as sigmoids. See also Tables S1 and S2 and Figure S1.

We recorded from 1840 sites in the primary auditory cortex (A1) and anterior auditory field (AAF) of eight anesthetized ferrets, while diotically presenting dynamic random chord (DRC) sequences. The chords were changed within each sequence every 25 ms, with the levels of their constituent tones (1/6 octave spaced) drawn from uniform distributions in SPL space. The contrast of the sequences was manipulated by changing the (SPL) standard deviation (*σ_L_*) of these distributions. The tone level distributions had identical mean (*μ_L_* = 40 dB SPL) but different widths: ± 5 dB (low contrast; *σ_L_* ≈2.9 dB, *c* = *σ_P_/μ_P_* = 33%), ± 10 dB (medium contrast; *σ_L_* ≈5.8 dB, *c* = *σ_P_/μ_P_* = 63.8%), or ± 15 dB (high contrast; *σ_L_* ≈8.7 dB, *c* = *σ_P_/μ_P_* = 91.6%) (Figure 1). The close relationship between contrast in sound pressure (*σ_P_/μ_P_*) and *σ_L_* for these distributions is shown in Figures S1A and S1B; these, together with other stimulus statistics, are documented in Table S1. As these distributions are primarily defined in SPL space, and as we performed analyses on units' stimulus-response relationships using stimulus representations in SPL space, we present our data and models here in terms of *σ_L_* rather than *σ_P_/μ_P_*, so as not to mix together the sound pressure and level domains.

The RMS sound level of the total stimulus ranged from 70 to 80 dB SPL. We identified 1001 units that responded reliably to the DRCs, as measured via a maximum noise level criterion (see Experimental Procedures).

Although the anesthetized preparation allowed for precise control of stimulation and eliminated the possibility of attentional modulation, to confirm that the observations made under anesthesia apply in awake animals, we also presented the same stimuli through a free-field speaker to an awake, passively listening ferret and recorded spiking activity from 62 sites in A1 and AAF. We identified 19 units that responded reliably to the DRCs. We found no differences in the response characteristics of neurons in the two preparations and therefore combined these data in subsequent analyses.

### Stimulus Contrast Has Little Systematic Effect on Spectrotemporal Tuning

**Figure 2:**
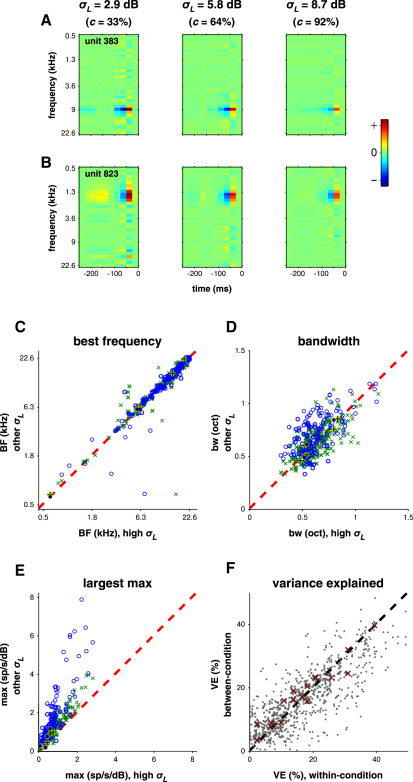
Effect of Stimulus Contrast on Tuning (A and B) Example units show the same spectrotemporal selectivity in the receptive fields (STRFs) estimated from the three contrast conditions. Red denotes components of the stimuli that excite the unit, blue denotes components that inhibit. As the color scale is uniform across the plots, it is clear that the dominant variation across the conditions lies in the magnitude of the drive to the unit: this is stronger (the cortex appears to “listen harder”) under low-contrast than under high-contrast stimulus conditions. (C–E) Best frequency (C) does not vary systematically with stimulus contrast across units. Tuning bandwidth (D) shows a small, significant broadening at low contrast. In almost all units, the gain of the STRF (E) increases as contrast decreases. STRF properties under low-contrast stimulation are shown as blue circles, under medium contrast as green crosses. Filled dark blue circles and dark green pluses indicate data in these two same conditions from the awake recordings. (F) The linear STRFs fitted from DRCs with different contrasts are sufficiently similar in tuning properties that, once adjusted for differences in STRF gain, they predict responses across stimulus conditions on average 96.5% as well as they do within their own conditions. Thus, most of the contrast dependence of STRFs is captured by a change in gain. Red crosses indicate data from the awake recordings. See also Table S3.

We first asked whether the tuning of cortical neurons is affected by changes in stimulus contrast. If this were the case, it would not be appropriate to describe such a response as gain control. We characterized the tuning of each unit by estimating one STRF for each contrast condition (e.g., Figure 2A; see Model 1 in Table S2). Only STRFs that had predictive power (see Experimental Procedures) were included in the further analysis; generally, the prediction scores were worse under lower-contrast stimulation (Table S3).

Changing stimulus contrast produced only small changes in STRF shape (Figures 2C and 2D). Of 261 units with predictive STRFs, 223 maintained the same best frequency (BF) across conditions (within 1/6 of an octave; Figure 2C). Twenty-six units had STRFs that were too diffuse to give clear BF estimates. Only 12 units showed evidence of changes (≤1/3 octave) in BF across conditions. Tuning bandwidths were slightly broader under low-contrast stimulation (sign-rank test; p << 0.001); however, this may reflect the noisier estimates of STRF coefficients at low contrast. Tuning bandwidth did not change systematically between medium- and high-contrast regimes (p > 0.5) (Figure 2D). We also observed no systematic changes in the temporal structure of STRFs, though this was limited by the 25 ms time resolution of the analysis.

To assess the importance of any unmeasured STRF shape changes, we modeled each neuron by a single linear STRF multiplied by a variable gain factor (Model 2 in Table S2). STRFs from one stimulus condition predicted responses in the other conditions as well as the within-condition STRFs (Figure 2F), indicating that any shape changes in the STRFs were negligible. Thus, auditory cortex neurons exhibit similar spectrotemporal preferences regardless of contrast. This is similar to previous observations in the IC (Escabí et al., 2003), but different from the visual system, where contrast has a considerable effect on the temporal dynamics of neural responses (Mante et al., 2005).

### Increased Response Gain Partially Compensates for Lower Stimulus Contrast

**Figure 3:**
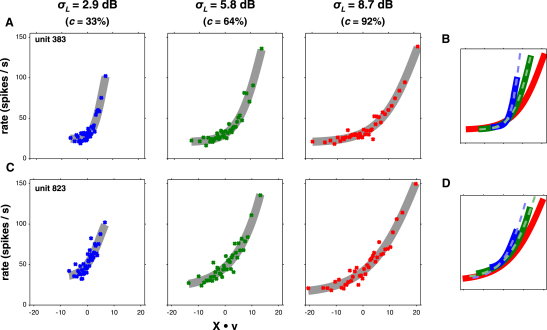
Output Nonlinearities for Two Example Units Show Gain Rescaling as a Function of Stimulus Contrast (A) For each unit, we fitted a single linear STRF, then calculated a separate output nonlinearity for each contrast condition. The abscissa denotes the output of the linear STRF, X⋅v; the ordinate is the predicted spike rate. (B) The differences between the nonlinearities were quantified by the linear transform required to convert the high-contrast nonlinearity into each of the medium- and low-contrast nonlinearities. Solid curves show the original sigmoids for the unit shown in (A); dashed lines show the result of the transformation of the high-contrast (red) curve into the low-contrast (blue) and medium-contrast (green) curves. The parameters for the high-to-low transform for this unit were *G* = 2.9, Δ*x* = 17.6%, Δ*y* = 8.9%; the high-to-medium transform parameters were *G* = 1.5, Δ*x* = 2.0%, Δ*y* = −0.6%. (C and D) Nonlinearities for a second example unit; panels equivalent to (A) and (B). Parameters for these transforms were *G* = 1.5, Δ*x* = −27.6%, Δ*y* = 2.4% (high-to-low) and *G* = 1.2, Δ*x* = −3.8%, Δ*y* = 8.5% (high-to-medium). See also Figure S2.

We observed substantial changes in gain between conditions, as measured by comparing the largest-magnitude coefficients of the STRFs (Figure 2E). To characterize gain changes more accurately, we extended the simple linear model to a LN one (Figures 1G and 3; Equation 5; Model 3 in Table S2). This comprised a single linear STRF for each unit, estimated from its responses across all conditions, followed by a sigmoidal output nonlinearity. Separate nonlinearities were fitted for each contrast condition. The LN model far outperformed the linear models: prediction scores were a median 38.5% higher than the within-condition linear models (p << 0.001; sign-rank). We found 315 units where LN models were predictive in all three contrast conditions. Analyses on the remaining units are presented in Figure S3G, showing results similar to those presented below.

In an LN model, differences in gain manifest through changes in the shape of the output nonlinearity. To quantify these changes, we calculated the set of linear transformations required to map the output nonlinearity for high-contrast stimulation (*σ_L_* = 8.7 dB, *c* = 92%) onto those for other stimulus conditions. In principle, this mapping could combine a scaling of the curve along the horizontal and vertical axes and a translation of the curve along these axes (x- and y-offset, respectively). However, none of the units under investigation operated near their saturation point, making an estimate of vertical scaling difficult. Thus, we measured changes in the remaining three degrees of freedom (Equation 6; Model 4 in Table S2). Horizontal scaling corresponds to a change in gain, x-offset to a threshold shift and y-offset to a change in minimum firing rate.

**Figure 4:**
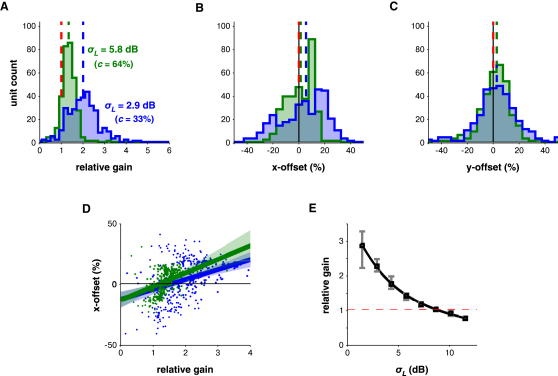
Output Gain Is Inversely Related to Stimulus Contrast (A) Histogram of gain (relative to the reference condition) under low- (blue) and medium-contrast (green) conditions, for n = 315 units. Colored dashed lines are population medians. Red dashed line indicates *G* = 1, i.e., no scaling of the nonlinearity. (B) As in (A), for changes in x-offset. Positive values denote rightward shifts in nonlinearities. (C) As in (A), for changes in y-offset. Positive values denote upward shifts in nonlinearities. (D) For both the high-to-low-contrast (blue) and the high-to-medium-contrast (green) transformations, changes in gain (abscissa) and x-offset (ordinate) were positively correlated across the population of units. Shaded regions show the (bootstrapped) 99% confidence intervals about the mean regression line (solid). (E) The population median gain over a wide range of contrasts was well modeled by a standard gain normalization function, Equation 2. Gray bars indicate 99% confidence intervals on the median, via bootstrapping. See also Figure S3.

We observed a robust relationship between stimulus contrast and gain across the population of units. An approximately 3-fold decrease in contrast from 8.7 dB (*c* = 92%) to 2.9 dB (*c =* 33%) increased gain by a median factor of 2.01; for an ∼1.5-fold decrease in contrast from 8.7 dB (*c* = 92%) to 5.8 dB (*c* = 64%), gain increased by 1.34× (Figure 4A). The gain effect was also strongest among units with the most robust, repeatable spike trains (Figure S3D). Gain therefore changes in the appropriate direction to compensate for changes in stimulus contrast, but this compensation is not complete.

Decreasing stimulus contrast also caused nonlinearities to shift by a small amount to the right (median x-offset of 5.5% and 1.4% for low and medium contrast; p < 0.001 and p < 0.05, respectively, sign-rank test; Figure 4B), but there was no corresponding vertical translation of these curves (Figure 4C). Although the change in x-offset is nominally indicative of a small increase in threshold, the gain and x-offset measures were correlated with each other across units (r^2^ = 0.195 in high-to-low- and 0.11 in high-to-medium-contrast curve transformations; Figure 4D), suggesting that the rightwards shift in curves partly acts to compensate for gain (see Figure S3E). The lack of systematic y-offset changes indicated that minimum firing rate did not change across conditions. Therefore, the primary consequence of decreasing stimulus contrast is that cortical cells increase their gain.

By transforming output nonlinearities across conditions, we could predict neural responses to each contrast stimulus as successfully as by using separate nonlinearities for each condition as described above (median difference in prediction scores of 0.7%; sign-rank, p > 0.5).

These effects are similar to the changes in coding accuracy previously observed in the IC (Dean et al., 2005). Neuronal firing is most sensitive to and hence most informative about stimulus changes when the slope of the input/output function is at its greatest. This occurs at a median position of X⋅v = 5.3, 10.1, and 14.3 under low, medium, and high contrast, respectively (Figure S2). These lie at approximately the same percentile (∼70%) of each stimulus distribution, relative to their projection onto X⋅v. Neurons in auditory cortex thus adapt their sensitivity to be most informative about stimuli within the current stimulus distribution.

To fully quantify the relationship between stimulus contrast and gain, we presented to a subset of these cells a larger set of DRCs with eight different *σ_L_* values ranging from 1.4 dB to 11.5 dB (*c* = 17% to 116%). We obtained 80 units for which the above analysis could be performed over the whole contrast range. On average, these showed a clear, monotonic increase in gain as the contrast of the stimulus was reduced (Figure 4E). The relationship between relative gain and contrast was extremely well described by a standard normalization equation (Heeger, 1992; Carandini et al., 1997):

(2) $$G\left(\sigma_{L}\right)=\frac{a}{1+b\;\sigma_{L}^{n}}$$

where *G* denotes the gain and *a*, *b*, and *n* are constants (see Model 5 in Table S2). This model explained 99.9% of the variance in the population average of relative gain values.

This model also provided a good description of the relative gain values for individual units (Figure S3H). However, in some units, the model failed at the lowest contrasts. For these units, gain increased as contrast was reduced down to a threshold, below which gain either leveled off or decreased. For 46/80 units, this threshold was *σ_L_* = 2.9 dB (*c =* 33%); for a further 26 units, this threshold was 4.3 dB (*c* = 49%); and a further four units had a threshold of *σ_L_* = 5.8 dB (*c =* 64%). At these thresholds and above, gain was well fit on a cell-by-cell basis by Equation 2 for 76/80 units. The model produced marginally better predictions of neural responses than fitting individual nonlinearities to each contrast condition (Table S2). Thus, across a wide range of contrasts, gain normalization is a robust phenomenon for individual units.

### Contrast Gain Control Is Weak at Low Mean Levels and Saturates at High Mean Levels

In the experiments presented so far, the mean SPL of each tone in the DRC, *μ_L_*, was kept fixed. To explore the effect of mean, we presented a further set of stimuli in which both the mean of the level distributions (*μ_L_*) and the contrast (*σ_L_*) were manipulated independently. We estimated LN models from responses to a range of mean/contrast conditions, together with curve transformations from each stimulus condition relative to the *μ_L_* = 40 dB SPL, *σ_L_* = 8.7 dB (*c =* 92%) nonlinearity. Of the 1001 units above, 56 units yielded predictive LN models across the whole range of conditions. Only data from these 56 units are analyzed below, in order to maintain the same sample set across stimulus conditions. Nevertheless, data from all units where LN models were predictive in only a subset of conditions (n = 217) yielded similar results (data not shown).

**Figure 5:**
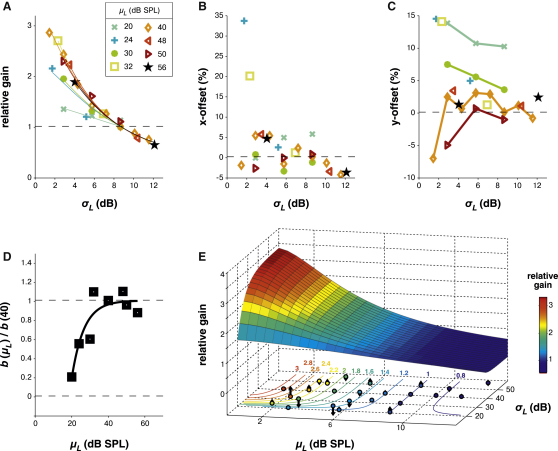
Contrast Gain Control Is Modulated by *μ_L_* Only at Low Mean Levels (A) Sigmoid nonlinearities were fitted to units' responses to a range of DRCs with different contrast and *μ_L_* statistics and compared with a reference curve. Population median gain factors are plotted against contrast, with different symbols/colors for each mean level. Colored lines show independent fits of the model in Figure 4E. (B) As in (A), for x-offset. (C) As in (A), for y-offset. (D) Values of *b* fitted to the model given in Equation 8, as shown in (A). These measure the relative sensitivity of neural gain to the stimulus contrast, as a function of mean level. At mean levels ≥35 dB SPL, $b\left(\mu_{L}\right)$ is relatively independent of *μ_L_*, while it becomes sensitive to *μ_L_* at lower mean levels. Solid line denotes an exponential fit to $b\left(\mu_{L}\right)$. (E) Illustration of the full model $G\left(\sigma_{L},\mu_{L}\right)$ for gain normalization (colored surface). A contour plot of this surface is projected below. Colored dots on the contour plot show the population median data used to constrain the model, from (A). Their position is a function of both *σ_L_* and *μ_L_*; their color denotes the median measured gain in these stimulus conditions; vertical arrows denote the residual between the measured gain and that described by the model. See also Figure S4.

At all mean levels tested, decreasing contrast caused gain to increase across the population of cells. However, the degree of gain normalization depended on the mean level: at low *μ_L_*, reducing contrast did not yield as much compensatory gain change compared with reducing contrast at high *μ_L_*. While increasing *μ_L_* therefore increased the dependence of gain on contrast, this trend saturated above *μ_L_* ≈35 dB SPL (Figure 5A). At higher mean levels, gain was decoupled from the mean sound level and varied with contrast alone. Interestingly, although changing mean level had no systematic effect on x-offset in our data (Figure 5B), reducing the mean level typically increased y-offset, i.e., raised the minimum firing rate (Figure 5C; examples in Figures S4A and S4B).

Given the success of Equation 2 in modeling the relationship between *σ_L_* and gain, we extended this model to include mean level, *μ_L_*. The most explanatory model (Equation 8) was a simple extension of the contrast-dependent model where *b* could vary with *μ_L_*. This allows *μ_L_* to directly modulate the dependence of gain on contrast. Fitted values for $b\left(\mu_{L}\right)$ are presented in Figure 5D, showing that at low *μ_L_*, *b* is modulated by *μ_L_*, whereas *b* saturates with high *μ_L_*. For simplicity, we modeled this with an exponential function (Equation 8; see also Model 6 in Table S2). This model explained 97% of the total variance in the data set (Figure 5E). We did not estimate the parameters for individual units, and therefore did not cross-validate this model.

All of the above results remained unchanged when gain was expressed as a function of *σ_P_*/*μ_P_* rather than *σ_L_* (Figure S4C).

### Responses to a Fixed Sound Depend on Context

The above results suggest that the recent spectrotemporal statistics of the stimulus modulate neural responses to a sound. We predicted that if a particular sound was presented in a low-contrast context, it would generate stronger responses than if presented in high-contrast context.

**Figure 6:**
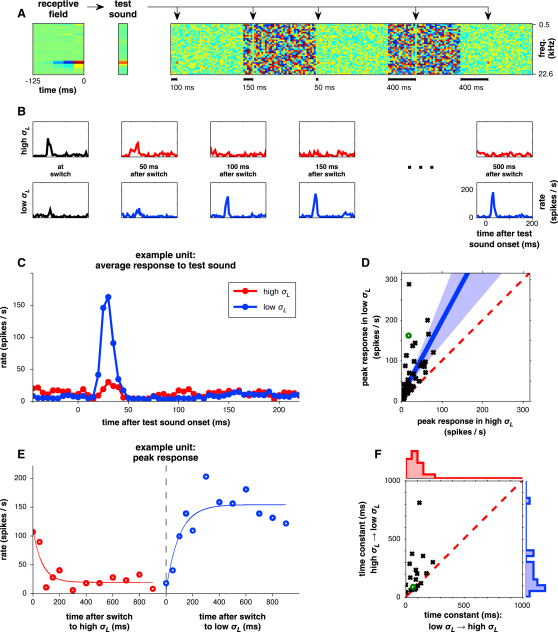
Responses to Fixed Sounds Are Modulated by the Spectrotemporal Contrast of their Context (A) During each electrode penetration, the STRF of a representative unit was used as a basis for a test sound. This was inserted at random times into special DRCs in which stimulus contrast switched every 1 s between low (*σ_L_* = 2.9 dB, *c =* 33%) and high contrast (*σ_L_* = 8.7 dB, *c =* 92%). The test sound itself was identical within each stimulus regime; only the contrast of its context differed. (B) Mean response to the test sound for an example unit, when presented in high-contrast (top row) or low-contrast context (bottom row). Columns delineate responses by the time since the last switch in context at which the test sounds were presented. (C) Response to the test sound for the unit in (B), averaged within each contrast context over all postswitch delays from 150–800 ms. (D) Peak responses to the test sound across n = 63 units, during the low- and high-contrast contexts. Red dashed line shows expected response relationship if contrast-context was irrelevant. Green circle indicates the unit in (B) and (C). Shaded region shows the confidence intervals as in Figure 4D. (E) Peak response for unit in (B) and (C) as a function of the time after context switch at which the test sound was presented. Solid lines show exponential fits to these data, with time constant *τ_L→H_* = 62 ms after an increase and *τ_H→L_* = 85 ms after a decrease in the contrast of the context. (F) Time constants for context adaptation, as in (E), for 18 units for which both *τ_L→H_* and *τ_H→L_* could be reliably estimated. Data are plotted both as a scatter plot and as marginal histograms of *τ_L→H_* (red) and *τ_H→L_* (blue). Green circle denotes unit in (B), (C), and (E). See also Figure S5.

To test this prediction, we embedded a fixed “test sound” into DRC segments of differing contrasts. This sound was designed to drive all units within an electrode penetration, by having stimulus energy within the receptive fields of the units recorded there (Figure 6A). The different contexts were provided by a DRC sequence that alternated between high (*σ_L_* = 8.7 dB, *c* = 92%) and low contrast (*σ_L_* = 2.9 dB, *c =* 33%) every 1 s. The same test sound was presented once per 1 s block at a random time relative to the onset of that block, i.e., the last switch in context. Among 63 units that responded reliably to the test sound, all but two responded more vigorously when this sound was presented in a low-contrast context than in a high-contrast context; the firing rate was a median 2.6 times greater in low-contrast context (p ≪ 0.001, sign-rank; Figures 6B–6D). This confirmed our prediction.

This experiment also allowed a finer-grained comparison of the time course of responses in high and low context. Similar to the STRF analysis, we found no systematic difference between these (Figure S5).

The variable timing of the test sound relative to the time of context switch allowed us to estimate the time course of the adaptation to stimulus contrast (Figure 6E). This could, in turn, inform a time-dependent model of gain control (e.g., Model 7 in Table S2), though we did not cross-validate such a model. Reliable estimates of time constants were obtained for both the switch from low- to high-contrast context (*τ_L→H_*) and the switch from high- to low-contrast context (*τ_H→L_*) for 18 units. Adaptation to high-contrast context occurred with a median *τ_L→H_* of 86 ms, compared with a slower adaptation to low-contrast context with a median *τ_H→L_* of 157 ms. This difference was significant (p < 0.001, sign-rank) and evident for 14/18 of the individual units (Figure 6F). Thus, the time courses for increases and decreases in neural gain are asymmetric.

### Neuronal Gain Is Dependent on Stimulus Contrast both Within and Outside the Tuning Curve

**Figure 7:**
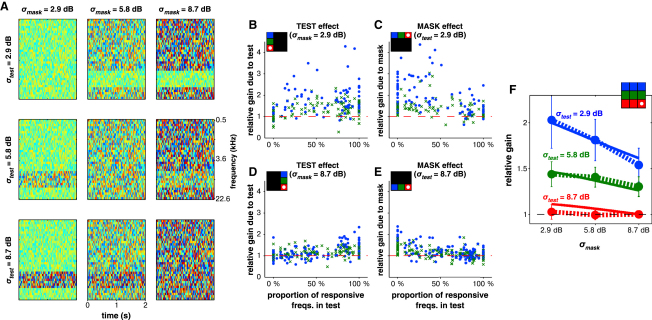
Gain Is Affected by the Contrast of Stimuli Lying both Within and Outside the Tuning of a Unit (A) During each electrode penetration, the STRF of a representative unit was mapped and the BF determined. A 0.5–1.2 octave band centered around the BF was designated the test and the remainder the mask. The contrast within these (*σ_test_*, *σ_mask_*) was independently varied. The example stimuli shown here were used for a unit with a BF of 9 kHz. (B–E) Relative gain from varying either *σ_test_* or *σ_mask_*, with the other kept constant. Color grids above each plot illustrate which stimulus conditions from (A) are being compared; the red box with the white dot is the reference curve used to calculate the transform. (F) Population median gain for 24 units with their responsive frequency range lying within the test (dots with dashed lines). Error bars denote 99% confidence interval on the median. Contrast within the test is a stronger determinant of the gain than that in the mask. Solid lines show the fit of Equation 3 to these data. See also Table S4.

To explore the mechanism for gain control, we asked whether gain is modulated by the contrast within a local region of frequency space or whether it is a function of the global statistics of the input. To address this, we varied the contrast of the DRC stimuli within two separate frequency regions. One region was denoted the “test,” centered around a chosen unit's BF and spanning 0.5, 0.67, or 1.2 octaves. The remaining frequency bands were denoted the “mask” (Figure 7A). We aimed to situate the test stimulus over the “responsive frequency range” ($\Phi_{RF}$; see Experimental Procedures), the frequencies to which a given neuron (linearly) responded. However, since we recorded multiple units simultaneously (usually bilaterally), we actually sampled a range of conditions where the test stimulus covered the neuron's responsive frequency range, overlapped it, or lay entirely outside it. This enabled us to measure how contrast gain depended on the amount of overlap between the test stimulus and $\Phi_{RF}$.

We presented nine separate DRCs, where the contrasts in the test (*σ_test_*) and mask (*σ_mask_*) were independently chosen from *σ_L_* = 2.9 dB, 5.8 dB, or 8.7 dB (*c =* 33%, 64%, or 92%). We found that the gain of each neuron was most strongly modulated by contrast within the responsive frequency range. Thus, varying *σ_test_* had the strongest effect on gain when the test stimulus completely covered $\Phi_{RF}$ (Figure 7B). Similarly, varying *σ_mask_* had the strongest effect when the mask completely covered $\Phi_{RF}$ (Figure 7C).

However, contrast away from the responsive frequency range also had an impact on gain. For example, even when the test stimulus completely covered $\Phi_{RF}$, decreasing *σ_mask_* still resulted in an increase in gain (Figure 7C). There were also interactions between contrast within and outside $\Phi_{RF}$ (compare Figure 7B with 7D and Figure 7C with 7E). This is summarized in Figure 7F for 24 units where the test completely covered $\Phi_{RF}$.

To quantify these effects, we extended the (*μ_L_*-independent) model (Equation 2) to include contributions to gain normalization from stimulus statistics both within and outside the responsive frequency range (“RF” and “global,” respectively):

(3) $$G\left(\sigma_{RF},\sigma_{global}\right)=\frac{a}{1+b_{RF}\sigma_{RF}^{n}+b_{global}\sigma_{global}^{n}}$$

Using only those units where the test covered their responsive frequency range, we fitted the model in Equation 3 assuming *σ_RF_* = *σ_test_*. This fit estimated a 2.4× stronger weighting of local stimulus contrast over global contrast (Model 8 in Table S2; Table S4) and captured the asymmetric interactions between *σ_test_* and *σ_mask_* (Figure 7F). In turn, the model was also successful at predicting the gain exhibited by units whose $\Phi_{RF}$ only partially overlapped the test or lay completely outside it (Table S4). The predictive value of this model points to either the existence of a gain control mechanism that strongly weights local over global stimulus statistics or else to the presence of two stages of gain control: one local and one global.

## Discussion

Our data show that the gain of neurons in auditory cortex is dynamically modulated according to the spectrotemporal statistics of recently heard sounds. The primary determinant of gain is stimulus contrast, which is well approximated by the standard deviation of the SPL (*σ_L_*). Gain decreases as stimulus contrast increases, thereby partially compensating for changes in contrast. Mean stimulus level also influences gain: when the mean level is low, the effectiveness of contrast gain control is reduced.

### Mechanisms for Gain Control

Our data focus on the effects of gain control, rather than on its specific implementation. Thus, although our models refer to stimulus contrast and level, we do not know how (or even whether) these parameters are explicitly computed by the brain. Nevertheless, by investigating how the gain signal depends on the spectral and temporal integration of stimulus statistics, we obtain insight into the mechanisms underlying gain changes. We find that gain is mainly determined by spectrotemporal contrast near the preferred frequency of each neuron, but there is also a significant contribution from the contrast outside the neuron's STRF (Figure 7). The time course of gain changes is asymmetric (Figure 6): time constants for increases and decreases in gain are 157 ms and 86 ms, respectively.

The observation that gain is regulated through wide spectral integration places some constraints on possible mechanisms. This suggests that gain control is not mediated entirely by a within-neuron mechanism, since single neurons do not have access to all the information required to calculate spectrotemporal contrast and adjust gain accordingly. This, along with the time course of gain changes, potentially argues against synaptic depression (Carandini et al., 2002), which could, in principle, operate much faster. It may, however, be necessary to integrate information over a number of successive stimuli before gain can be adjusted in this fashion; this argument incidentally provides a computational justification for the asymmetry in adaptation times (DeWeese and Zador, 1998). The influence of distant frequent bands is also unlikely to result from masking in the auditory periphery: although higher spectrotemporal contrast produces larger variation in the level of each frequency band, the total level of the DRCs remains relatively constant both over time and between conditions.

Alternative possibilities are that gain control is mediated by an intracortical network (Carandini et al., 1997) or through cortico-thalamic feedback, via recurrent excitation and inhibition (e.g., Abbott and Chance, 2005). Both hypotheses are compatible with the spectral and temporal integration we find here. Nevertheless, it is likely that gain control in cortex is at least partly inherited from earlier auditory structures. It has been shown, for example, that responses of neurons in the mammalian IC (Kvale and Schreiner, 2004; Dean et al., 2005, 2008) alter their gain to compensate for the temporal contrast of the level of a noise stimulus. The time constants of these effects are similar to those we observe in cortex and show a similar asymmetry for increases and decreases in gain. If the mechanisms in cortex and midbrain are identical, we would expect gain modulation in the IC to show the same spectral spread as we observe here. Characterization of both the spectral and temporal properties of gain control is likely to be informative in either linking or distinguishing between gain effects in cortex and more peripheral stations, such as those observed by varying the modulation depth of sinusoidally amplitude-modulated tones in the auditory nerve (Joris and Yin, 1992) or by varying the spectral contrast of complex chords in the brainstem (Reiss et al., 2007).

Finally, there may be a number of independent gain control stages at different levels of the auditory system. These may have different characteristics and time constants, reflecting different underlying mechanisms. Such a hierarchy has been observed in the visual system, where at least both the retina and V1 engage separate gain control mechanisms (Carandini et al., 1997; Brown and Masland, 2001; Chander and Chichilnisky, 2001; Baccus and Meister, 2002). In the extreme, gain control may be performed at every stage along the pathway (for review, see Kohn, 2007). If there are multiple, independent stages of gain control, then the local (within-receptive-field) gain effects and the global (extra-receptive-field) gain effects may be realized by different mechanisms and at different levels of the pathway. Further experiments will be required to distinguish these components by separately measuring their spectral and temporal parameters.

If distinct local and global mechanisms are involved, perhaps with different time courses, then synaptic depression could still be a strong candidate mechanism for the local mechanism, as it has been implicated in gain control across a broad range of neural systems (Stratford et al., 1996; Carandini et al., 2002; Chung et al., 2002). Within the auditory system itself, forward suppression—whereby the response of neurons to a sound is reduced when another sound precedes it—lasts for >100 ms in A1, which corresponds to a suppression of synaptic conductances (Wehr and Zador, 2005) or activation of hyperpolarizing currents (Abolafia et al., 2011). Synaptic depression also shows temporal asymmetry similar to that observed here (Hosoya et al., 2005; Dobrunz et al., 1997; Chung et al., 2002).

### The Role of Contrast Gain Control

Gain control is primarily useful for adapting the limited dynamic range of a neuron to the statistics of the stimulus. When spectrotemporal contrast is low, firing rates are sensitive to smaller changes within their spectral “region of interest” than under higher-contrast conditions. Thus, the representation of stimulus space is effectively expanded under low-contrast stimulation and compressed under high-contrast stimulation. Consequently, gain control should improve the ability of individuals to detect small changes in low-contrast sounds. Indeed, a related phenomenon has been demonstrated in the adaptation to reverberation, whereby listeners are better able to discriminate (low-contrast) reverberant words when embedded within a reverberant context sentence than within a (high-contrast) anechoic context (Watkins, 2005), an effect that is also frequency-band specific (Watkins and Makin, 2007). Perceptual adaptation is not, however, complete, as a general increase in the spectrotemporal contrast of speech leads to demonstrable gains in intelligibility (Steeneken and Houtgast, 1980; van Veen and Houtgast, 1985; Miller et al., 1999). Our data predict that perceptual adaptation to stimulus contrast should be observable with nonspeech stimuli as well.

Neurons in the visual system are subject to contrast gain control, which is thought to be desirable for efficient coding of natural images (Schwartz and Simoncelli, 2001). Since the contrast of natural images is correlated across space and time, normalization by stimulus contrast reduces the redundancy of the neural code (Barlow, 1961; Vinje and Gallant, 2002). The contrast of a complex visual stimulus can be defined as *σ_I_*/*μ_I_*, which is strongly related to the two contrast measures that we have shown to determine auditory gain control (*σ_L_*, Figure 5A; *σ_P_/μ_P_*, Figure S4C). Auditory gain control may therefore have a similar redundancy-reducing effect. Although the ensemble (i.e., long time scale) distributions of natural sounds have been explored (Attias and Schreiner, 1997; Escabí et al., 2003; Singh and Theunissen, 2003), a deeper understanding of the relationship between contrast gain control and the statistics of natural sounds will require a characterization of natural sound level distributions at the temporal scales over which gain control operates.

We show that when stimulus level statistics are not uniform across the spectrum, gain control is also unevenly applied to neurons, depending on their frequency tuning. A spectrally limited band of high contrast has the greatest compressive effect on neurons if their tuning curves overlap this band. Conversely, neurons tuned to other frequencies maintain sensitivity to small changes in their input. Because natural sounds do not cover the entire audible frequency range evenly, such an arrangement might make it possible to match contrast adaptation to the challenges posed by each particular acoustic environment.

### Gain Control and Contrast Tuning

Although the gain change we observe is strong, it does not completely compensate for changes in stimulus contrast: even at high mean stimulus levels (where contrast gain control is most effective and independent of sound level), an approximately 3-fold reduction in spectrotemporal contrast yields only an ∼2-fold increase in gain. Thus, gain control does not result in contrast invariance. Indeed, previous studies (Barbour and Wang, 2003; Escabí et al., 2003) have found that some auditory neurons are contrast tuned, firing more in response to some contrasts than others. Such a result would be incompatible with contrast invariance, but is compatible with the incomplete contrast compensation observed here. Taken together, these results suggest that auditory cortex uses both a division-of-labor strategy and adaptive gain control. Gain control reduces the range of stimulus values that must be separately encoded; within the remaining narrow range, a division-of-labor strategy may be used.

The incompleteness of gain control also suggests that there is a preferred range of stimulus contrasts for which neural coding is optimal; outside this range, gain control will fail to adjust gain enough to bring the stimuli into the neurons' dynamic range. It is possible that this preferred distribution is defined by the ensemble of natural sounds (Attneave, 1954; Barlow, 1961; Schwartz and Simoncelli, 2001; Lewicki, 2002).

It does not appear that gain normalization operates with equal measure from neuron to neuron. Not only does the strength of the effect differ across neurons, but only a subset continues to increase their gain as stimulus contrast is reduced to ever smaller levels (Figure S3H). This implies that different cortical neurons will be optimal encoders of different spectrotemporal level distributions. Similar diversity in adaptive properties has also been found in awake marmoset cortex, where subclasses of cells either adapt to the mean sound level of a stimulus or maintain a fixed preference for a particular intensity range (Watkins and Barbour, 2008). Just as such cells retain the ability to detect soft sounds in a loud environment, a variation in the degree of gain normalization between neurons may help retain the ability to detect small changes in high-contrast environments. These are particularly important tasks in audition, where superimposed sound sources need to be detected and dissected.

Finally, given the strength of gain normalization observed in this study, we predict that including gain control will prove to be a generally important factor in improving the predictive power of STRF models of auditory processing. However, the implementation details may prove crucial. For instance, normalizing by global stimulus contrast, without taking into account spectrally local contrast, does not result in an improvement in the predictive power of STRF models (David et al., 2009). This suggests that a detailed implementation of the spectral and temporal integration that informs the gain signal, such as that initiated in this study, will be needed before such improvements can be made.

## Experimental Procedures

### Animals and Anesthesia

All animal procedures were approved by the local ethical review committee and performed under license from the UK Home Office. Eight adult pigmented ferrets (6 male, 2 female) were chosen for electrophysiological recordings under ketamine-medetomidine anesthesia. Extracellular recordings were made using silicon probe electrodes (Neuronexus Technologies, Ann Arbor, MI) with 16 sites on a single probe, vertically spaced at 50 μm or 150 μm. Stimuli were presented via Panasonic RPHV27 earphones (Bracknell, UK), coupled to otoscope specula that were inserted into each ear canal, and driven by Tucker-Davis Technologies (Alachua, FL) System III hardware (48 kHz sample rate). Further recordings were made in an awake, passively listening female ferret, with free field stimulation presented in an anechoic room via an Audax TWO26M0 speaker (Audax Industries, Château du Loir, France) ∼80 cm from the animal's head. Full experimental procedures are described in Bizley et al. (2010).

Offline spike sorting was performed using spikemonger, an in-house software package (see Supplemental Experimental Procedures). We included only units that showed acoustically responsive activity.

### Dynamic Random Chords

The main stimulus was a DRC: a superposition of 34 pure tones, with frequencies log-spaced between 500 Hz and 22.6 kHz at 1/6 octave intervals. The tone levels during each chord were independently drawn from a uniform distribution, with mean level *μ_L_* (dB SPL). The distribution was uniform across (logarithmic) level, not (linear) RMS pressure, as this better matches the range of sound intensities and modulations present in natural signals (Escabí et al., 2003; Gill et al., 2006). The distribution width was varied, giving three stimulus contrasts (Figure 1). For a subset of recordings, a broader range of widths was presented (from ±2.5 dB to ±20 dB in 2.5 dB steps). A full range of stimulus statistics is given in Table S1.

Chords were 25 ms in duration and presented in sequences of 15 s or 30 s duration. The overall RMS level of the stimuli was 71.0 ± 0.5 dB SPL in low contrast, 72.4 ± 1.0 dB SPL in medium contrast, and 74.5 ± 1.5 dB SPL in high contrast, when *μ_L_* = 40. A control experiment was performed to show that these small differences in the overall level did not account for gain control (data not shown).

To build the sequences, we first generated random levels for each tone in each chord. A new random seed was used for each electrode penetration and stimulus condition. We synthesized each tone, applied envelopes based on the random levels (with 5 ms linear ramps between chords), and then superimposed them. This ensured there were no amplitude or phase discontinuities in the signal. Each DRC sequence was presented 5–20 times (10 times for the awake animal), randomly interleaved, with 15–20 s silence between each sequence. The first 2 s of data from each presentation were discarded to ensure that a constant adaptation state had been reached.

### Signal Power and Noise Power

Since the analyses carried out here can only be applied to acoustically driven units that produce reasonably reliable, repeatable responses, we calculated the noise ratio (NR) for the PSTHs of each unit (Sahani and Linden, 2003b):

(4) $$\text{noise}\;\text{ratio}\;=\;\frac{\text{noise}\;\text{power}}{\text{signal}\;\text{power}}\;=\;\frac{\text{total}\;\text{variance}-\text{explainable}\;\text{variance}}{\text{explainable}\;\text{variance}}$$

An NR of 0 indicates that responses were identical for repeated stimulus presentations. Higher NR indicates that responses are less reliable. Units with NR > 10 in any one stimulus condition, i.e., whose explainable variance was <9.1% of the total variance, were excluded from further analysis. NRs were highest in the low-contrast condition (Table S3). Thus, we used data from the high-contrast condition as the reference for comparisons.

### STRF Estimation

STRFs were estimated by correlating the stimulus history with the spike peristimulus time histogram (PSTH). The PSTH was binned at 25 ms; bins were offset by between 0 and 25 ms to allow for response latency. The offset was chosen to minimize the NR. We estimated a separable kernel, $w_{ft}$, such that $w_{ft}=w_{f}⊗w_{t}$, where $w_{f}$ is the frequency kernel, $w_{t}$ the time kernel, and ⊗ the outer product, via maximum likelihood (Sahani and Linden, 2003a; Ahrens et al., 2008). Separable STRFs gave more accurate predictions than fully inseparable STRFs (which had more parameters). STRFs were trained on 9/10 of the available data for each unit and were used to predict a PSTH for the remaining 1/10. The prediction score is defined as the proportional reduction in the mean squared error of the response; if this was positive, the STRF was deemed predictive.

STRFs were estimated separately for each stimulus condition and for the pooled data set. The separate set of STRFs was used for the linear analysis (Figure 2); the pooled STRFs were used thereafter. In each case, units whose STRFs or LN models (see below) were not predictive on the validation data set were excluded from analysis.

The measurement of BF and bandwidth of each STRF is described in the Supplemental Experimental Procedures.

### Nonlinearities

We refined the linear STRF by fitting a LN model to units' responses (Chichilnisky, 2001). The STRF is a linear approximation of the relationship between the stimulus **X** and response *Y*, via Y=X⋅w+ɛ. To capture nonlinearities in this relationship, we fitted a nonlinear function to the output of the linear model, such that Y=F[X⋅v]+ɛ. Here, v=w/‖w‖ is the unit vector in the direction of the STRF, i.e., the direction of stimulus space to which the cell is (linearly) sensitive. *F* was approximated by dividing the stimulus/response pairs into 40 bins along the X⋅v axis and averaging responses within each bin. A logistic curve (sigmoid) was fitted to the data via gradient descent:

(5) $$F\left[X\cdot v\right]\;=\;b_{1}+\frac{b_{2}}{1+b_{3}\;\text{exp}\left(b_{4}\left(X\cdot v\right)\right)}$$

To check that pooling responses from different stimulus conditions in the initial STRF estimation was valid, we built LN models for each cell using STRFs estimated from only one stimulus condition. Results were similar, regardless of which condition was used to build the STRF (Figures S3A–S3C).

### Curve Transformations

Independent sigmoids were fitted to the responses from each contrast condition. To describe the differences between the sigmoids, we chose the nonlinearity for the *σ_L_* = 8.7 dB (*c* = 92%) condition for every unit as a reference and found the linear transformations required to map the reference sigmoid onto the sigmoids obtained under the other conditions (see main text). This amounts to solving the equation:

(6) $$F_{\sigma_{L}}\left[X\cdot v\right]=F_{\sigma_{0}}\left[g.\left(X\cdot v\right)+\Delta x\right]+\Delta y$$

where $\sigma_{0}=8.7$ is the reference condition, *g* is the horizontal scale factor (gain change), Δx is the x-offset, and Δy is the y-offset. Details of this fit are provided in the Supplemental Experimental Procedures. For a given unit, Δx is expressed as a percentage of the size of the domain of X⋅v in the reference condition for that unit, while Δy is expressed as a percentage of $F_{\sigma_{0}}\left[0\right]$.

### Test Sound

For a subset of electrode penetrations, the STRF of a representative unit was estimated online, and used to create a test sound. The frequency component of the STRF, $w_{f}$, was scaled to create a single chord of 25 ms duration, $X_{T}$, that roughly fit the statistics of a DRC segment with medium contrast (Figure 6A). A set of new DRCs was generated for that electrode penetration, consisting of 25 alternating 1 s segments of low (*σ_L_* = 2.9 dB, *c =* 33%) and high contrast (*σ_L_* = 8.7 dB, *c =* 92%). $X_{T}$ was inserted once into each segment, at a random delay after each segment transition. Forty sequences, with different random seeds and test sound timing, were presented. To ensure that the test sound actually drove all the units in a given electrode penetration, only those units for which $X_{T}\cdot v\;>\;10\;\text{dB}$ were retained for analysis. Responses to the test sound were averaged for each combination of context (contrast of the DRC segment) and timing (delay after transition) conditions.

To estimate response latency, we binned the spiking response to the test sound at 5 ms resolution, averaged over all conditions, and defined a 15 ms window about the peak of the PSTH. Spiking within this window was defined as the peak response, *r(t)*. For units whose peak responses satisfied a reliability criterion (see Supplemental Experimental Procedures), time constants for adaptation were estimated by fitting the equation r(t)=a+b.exp(−t/τ).

### Test/Mask

To assess whether neuronal responses depend on stimulus contrast both within and outside the frequency range of their STRFs, a subset of units were probed with a set of specially constructed test/mask stimuli.

During recording, units' STRFs and BFs were estimated. From the set of 34 tone frequencies used in the DRCs (Φ), tones in a “test” band of 7 frequencies ($\Phi_{test}$), spanning half an octave above and half an octave below the unit's BF, had levels drawn from a different distribution from those in the remaining “mask” frequency bands ($\Phi_{mask}$). Nine different stimuli (Figure 7A) were presented five times each, randomly interleaved. Some units' BFs lay in the 2–3 highest-frequency bands of the DRCs; for these units, the test band was reduced to a width of either 3/6 or 4/6 octaves. Results from these units were similar, and so results from all three cases were pooled. For all units, a linear STRF was calculated from the pooled data set, and individual nonlinearities were calculated for each stimulus condition.

The responsive frequency range of each unit ($\Phi_{RF}$) was defined by which components of $w_{f}$ were significantly nonzero, via bootstrapping (see Supplemental Experimental Procedures). We then defined the overlap between $\Phi_{RF}$ and test:

(7) $$\frac{\sum_{f_{i}\;\in \;\Phi_{RF}}\left|w_{f_{i}}\right|}{\sum_{f_{i}\;\in \;\Phi }\left|w_{f_{i}}\right|}$$

where $w_{f_{i}}$ denotes the component of $w_{f}$ corresponding to frequency $f_{i}$.

### Normalization Models

To model the effects of stimulus statistics on neural gain, we extended a well-known class of gain normalization equations used in the visual system, which take the general form of Equation 2. As all gain values were computed relative to a reference curve ($\sigma_{ref}=8.7\text{dB}$), we fixed $a=1+b\;\sigma_{ref}^{n}$ to constrain $G\left(\sigma_{ref}\right)=1$.

To model the effects of varying both *σ_L_* and *μ_L_*, we fitted separate values for *b* (and therefore for *a*) for each mean level:

(8) $$G\left(\sigma_{L},\mu_{L}\right)=\;\frac{a\left(\mu_{L}\right)}{1+b\left(\mu_{L}\right)\sigma_{L}^{n}}$$

where $a\left(\mu_{L}\right)=1+b\left(\mu_{L}\right)\sigma_{ref}^{n}$ so that $G\left(\sigma_{ref},\mu_{L}\right)=1$ for all *μ_L_* (as observed in the data); *n* is constant with respect to *μ_L_*. The fit obtained was slightly better than if *n* was allowed to vary as a function of *μ_L_* and *b* was kept constant with respect to *μ_L_*.

Following the empirical fitting of $b\left(\mu_{L}\right)$ values, *b* was parameterized using the form $b\left(\mu_{L}\right)=b_{max}\left(1-e^{-c\left(\mu_{L}+k\right)}\right)$ to capture the saturation of $b\left(\mu_{L}\right)$ at high *μ_L_*.

For the test/mask analysis, we fitted Equation 3 for units where the test completely covered their responsive frequency range, assuming that $\sigma_{RF}=\sigma_{test}$, *n* given from fitting Equation 2, and *a* constrained by $G\left(\sigma_{ref},\sigma_{ref}\right)=1$. As above, this gave slightly better fits than fixing $b_{RF}=b_{test}=b$ and using separate exponents for $\sigma_{RF}$ and $\sigma_{global}$. The fitted parameters were used with Equation 3 to predict the gain for units where the test only partially covered $\Phi_{RF}$ or lay outside of it. The local contrast in this region and the global contrast were then calculated via the weighted sums:

(9) $$\sigma_{RF}^{2}\;=\;\frac{1}{\left|\Phi_{RF}\right|}\sum_{f\;\in \;\Phi_{RF}}\sigma_{L}^{2}\left(f\right)$$

(10) $$\sigma_{global}^{2}\;=\;\frac{1}{\left|\Phi \right|}\sum_{f\;\in \;\Phi }\sigma_{L}^{2}\left(f\right)$$

where $\sigma_{L}\left(f\right)$ is the contrast in frequency band *f*.

Successive models used to fit the response and relative gain of neurons in this study, together with best fit parameter values, are summarized in Table S2. Further information on the test/mask model, including alternate fits, is provided in Table S4.

## Supplemental Information

Document S1. Four Tables, Five Figures, and Supplemental Experimental Procedures
