# Supplementary material for: Contrast Gain Control in Auditory Cortex
Source: Neuron. 2011 Jun 23;70(6):1178–91. doi: 10.1016/j.neuron.2011.04.030 (PMC3133688; doi:10.1016/j.neuron.2011.04.030)
Supplement: Document S1. Four Tables, Five Figures, and Supplemental Experimental Procedures [file mmc1.pdf]

Neuron, Volume *70*

Supplemental Information

Contrast Gain Control in Auditory Cortex

Neil C. Rabinowitz, Ben D.B. Willmore, Jan W.H. Schnupp, and Andrew J. King

Inventory:

Table S1 is related to Figure 1.

Table S2 is related to Figure 1.

Figure S1 is related to Figure 1.

Table S3 is related to Figure 2.

Figure S2 is related to Figure 3.

Figure S3 is related to Figure 4.

Figure S4 is related to Figure 5.

Figure S5 is related to Figure 6.

Table S4 is related to Figure 7.

Supplemental Experimental Procedures.

Table S1: Summary of stimulus statistics

tone level distribution			tone pressure distribution			(total) DRC level		
**mean** μ_L (dB SPL)	**halfwidth** w (dB)	**s.d.** σ_L (dB)	**mean** μ_P (mPa)	**s.d.** σ_P (mPa)	**contrast** $c = \frac{\sigma_P}{\mu_P}$ (%)	**min** (dB SPL)	**RMS** (dB SPL)	**max** (dB SPL)
**Constant mean level**								
40	± 2.5	1.4	2.0	0.3	17 %	69.9	70.8	71.5
40	± 5	2.9	2.1	0.7	33 %	69.4	71.1	72.6
40	± 7.5	4.3	2.3	1.1	49 %	69.1	71.7	73.8
40	± 10	5.8	2.5	1.6	64 %	69.0	72.5	75.2
40	± 12.5	7.2	2.8	2.2	78 %	69.1	73.4	76.7
40	± 15	8.7	3.1	2.9	92 %	69.4	74.6	78.3
40	± 17.5	10.1	3.7	3.8	104 %	69.8	75.8	80.1
40	± 20	11.5	4.3	5.0	116 %	70.4	77.2	82.0
**Varied mean level**								
20	± 5	2.9	0.2	0.1	33 %	49.4	51.1	52.6
20	± 10	5.8	0.2	0.2	64 %	49.0	52.5	55.2
20	± 15	8.7	0.3	0.3	91 %	49.4	54.6	58.3
24	± 3	1.7	0.3	0.1	20 %	53.8	54.8	55.7
24	± 9	5.2	0.4	0.2	58 %	53.0	56.1	58.6
30	± 5	2.9	0.7	0.2	33 %	59.4	61.1	62.6
30	± 10	5.8	0.8	0.5	64 %	59.0	62.5	65.2
30	± 15	8.7	1.0	0.9	91 %	59.4	64.6	68.3
32	± 4	2.3	0.8	0.2	26 %	61.6	62.9	64.2
32	± 12	6.9	1.1	0.8	75 %	61.1	65.2	68.4
48	± 6	3.5	5.4	2.1	39 %	77.3	79.3	81.1
48	± 18	10.4	9.5	10.1	107 %	77.9	84.1	88.5
50	± 5	2.9	6.7	2.2	33 %	79.4	81.1	82.6
50	± 10	5.8	7.8	5.0	64 %	79.0	82.5	85.2
50	± 15	8.7	10.0	9.1	91 %	79.4	84.6	88.3
56	± 7	4	14.0	6.4	45 %	85.2	87.6	89.6
56	± 21	12.1	29.1	35.0	120 %	86.6	93.8	98.8

	tone level distribution			tone pressure distribution			(total) DRC level		
	**mean** μ_L **(dB SPL)**	**half-width** w **(dB)**	**s.d.** σ_L **(dB)**	**mean** μ_P **(mPa)**	**s.d.** σ_P **(mPa)**	**contrast** $c = \frac{\sigma_P}{\mu_P}$ **(%)**	**min (dB SPL)**	**RMS (dB SPL)**	**max (dB SPL)**
**Test / mask**									
Test:	40	± 5	2.9	2.1	0.7	33 %	69.1	71.1	72.6
Mask:		± 5	2.9	2.1	0.7	33 %			
Test:	40	± 5	2.9	2.1	0.7	33 %	68.3	72.2	74.7
Mask:		± 10	5.8	2.5	1.6	64 %			
Test:	40	± 5	2.9	2.1	0.7	33 %	68.1	73.9	77.4
Mask:		± 15	8.7	3.1	2.9	92 %			
Test:	40	± 10	5.8	2.5	1.6	64 %	69.3	71.4	73.4
Mask:		± 5	2.9	2.1	0.7	33 %			
Test:	40	± 10	5.8	2.5	1.6	64 %	68.5	72.5	75.3
Mask:		± 10	5.8	2.5	1.6	64 %			
Test:	40	± 10	5.8	2.5	1.6	64 %	68.3	74.2	77.9
Mask:		± 15	8.7	3.1	2.9	92 %			
Test:	40	± 15	8.7	3.1	2.9	92 %	69.2	71.9	74.7
Mask:		± 5	2.9	2.1	0.7	33 %			
Test:	40	± 15	8.7	3.1	2.9	92 %	69.0	72.9	76.4
Mask:		± 10	5.8	2.5	1.6	64 %			
Test:	40	± 15	8.7	3.1	2.9	92 %	68.9	74.6	78.6
Mask:		± 15	8.7	3.1	2.9	92 %			

Tone level and tone pressure statistics have been calculated from the respective analytic distributions of these variables shown in Figures 1 and S1. Statistics of the total level of the DRC (last three columns of the table) have been estimated from sample DRCs of 30 s duration.

Table S2, related to Figure 1: Summary of models

**1. linear separate**	$\hat{y}_i = \mathbf{w}_{ft}(\sigma_L) \cdot \mathbf{x}_{ift}$<br>$\mathbf{w}_{ft} = \mathbf{w}_f \otimes \mathbf{w}_t$<br>$\mathbf{x}_{ift} = \mathbf{X}_{ift} - \mu_L$
parameters:	$\hat{y}_i$ = response in time block i<br>$\mathbf{w}_{ft}(\sigma_L)$ = separable STRF for each contrast condition<br>$\mathbf{w}_f$ = frequency component of STRF<br>$\mathbf{w}_t$ = temporal component of STRF<br>$\mathbf{X}_{ift}$ = stimulus level (dB SPL) in time block $i-t$ and freq. band f.<br>μ_L = stimulus mean level (dB SPL)
use:	Figure 2A-E
cross-validated:	Yes. STRFs trained on 9/10 of available data, and used to predict a PSTH on the remaining 1/10.

**2. linear scaled**	$\hat{y}_i = g_{\sigma_L} \cdot (\mathbf{v}_{ft} \cdot \mathbf{x}_{ift})$<br>$\mathbf{v} = \frac{\mathbf{w}}{\lVert\mathbf{w}\rVert}$
parameters:	g_{σ_L} = gain for each contrast condition<br>$\mathbf{v}_{ft}$ = normalized receptive field
use:	Figure 2F
cross-validated:	Yes. STRFs from one stimulus condition (together with the gain factor g_{σ_L}) predicted responses in the other conditions as well as the within-condition STRFs (median difference in prediction scores of 3.5%, P > 0.4; Figure 2F).

**3. nonlinear separate**	$\hat{y}_i = F_{\sigma_L}\left[\mathbf{v}_{ft} \cdot \mathbf{x}_{ift}\right]$<br>$F_{\sigma_L}[z] = a_{\sigma_L} + \frac{b_{\sigma_L}}{1 + c_{\sigma_L}\exp\left[d_{\sigma_L} z\right]}$
parameters:	F_{σ_L} = point nonlinearity for each contrast condition<br>$a_{\sigma_L}, b_{\sigma_L}, c_{\sigma_L}, d_{\sigma_L}$ = sigmoid parameters for each contrast condition
use:	Figure 3
cross-validated:	Yes. Nonlinearities trained on 9/10 of available data, and used to predict a PSTH on the remaining 1/10.

**4. nonlinear transformed**	$\hat{y}_i = F\left[g_{\sigma_L} \cdot \left(\mathbf{v}_{ft} \cdot \mathbf{x}_{ift}\right) + \Delta x_{\sigma_L}\right] + \Delta y_{\sigma_L}$
parameters:	g_{σ_L} = horizontal scale factor, i.e. gain<br>Δx_{σ_L} = x-offset<br>Δy_{σ_L} = y-offset<br>For $\sigma_L = \sigma_{ref} = 8.7\,\text{dB}$: $g_{\sigma_L} = 1,\ \Delta x_{\sigma_L} = 0,$ and $\Delta y_{\sigma_L} = 0$
use:	Figure 4A-C
cross-validated:	Yes. Across units, nonlinearities from the reference stimulus condition, together with the fitted curve transformation, predicted responses in the other conditions as well as Model #3 above (median improvement in prediction scores of 0.2%; P > 0.3 by sign-rank test).

**5. simplified nonlinear scaled**	$\hat{y}_i = F\left[G(\sigma_L) \cdot \left(\mathbf{v}_{ft} \cdot \mathbf{x}_{ift} + \beta\right)\right]$<br><br>$G(\sigma_L) = \dfrac{a}{1 + b\,\sigma_L^n}$
parameters:	a specified by $G\left(\sigma_{ref}\right) = 1$
fitted values:	$n = 1.31$, $b = 0.15$ (Fig. 4E though values of n between 1.2 to 1.8 gave almost equally good fits)<br>$\beta \approx 1.9$ (Fig. 4D)
use:	Figures 4E, S3H
cross-validated:	Yes. This model was fit on a unit-by-unit basis to all data for each unit (where $\mu_L = 40$ dB SPL), fixing $n = 1.31$. Predictions of units' responses in each contrast condition were marginally better than Model #3 above (median improvement in prediction scores of 0.3%, P < 0.01 by sign-rank test). For units where gain did not increase monotonically as contrast was reduced to its lowest value ("threshold"), small improvements in predictions were obtained by limiting this model to responses above threshold.

**6. μ / σ interaction**	$\hat{y}_i = F\left[G(\sigma_L, \mu_L) \cdot \left(\mathbf{v}_{ft} \cdot \mathbf{x}_{ift} + \beta\right)\right] + H(\mu_L)$<br><br>$G(\sigma_L, \mu_L) = \dfrac{a(\mu_L)}{1 + b(\mu_L)\,\sigma_L^n}$<br><br>$b(\mu_L) = b_{max}\left(1 - e^{-c(\mu_L + k)}\right)$<br><br>$H(\mu_L) \approx h\,\mu_L$
parameters:	$a(\mu_L)$ specified by $G\left(\sigma_{ref}, \mu_L\right) = 1$ for all μ_L<br>$n = 1.31$ inherited from #5 above

fitted values:	$b_{max} = 0.17, c = 0.11, k = -18.6$ dB (Fig. 5D)<br>$h \approx -0.35$ %/dB (Fig. 5C)
use:	Figure 5
cross-validated:	No

**7. time-varying**	$G(\sigma_L) = G(\sigma_{L,i})$<br><br>$\sigma^2_{L,i} = A \,.\, \sum_{t=0}^{\infty} e^{-t/\tau} \left(\sum_f x^2_{ift} \right)$
parameters:	τ = time constant of adaptation<br>A = normalization constant
fitted values:	$\tau \approx 120\,\text{ms}$ (Fig. 6F), though this is asymmetric for increases and decreases in contrast
use:	Not implemented
cross-validated:	No

**8. test/mask**	$G(\sigma_{RF}, \sigma_{global}) = \frac{a}{1 + b_{RF}\,\sigma^n_{RF} + b_{global}\,\sigma^n_{global}}$<br><br>$\sigma^2_{RF} = \frac{1}{\lvert \Phi_{RF} \rvert} \sum_{f \in \Phi_{RF}} \sigma^2_L(f)$<br><br>$\sigma^2_{global} = \frac{1}{\lvert \Phi \rvert} \sum_{f \in \Phi} \sigma^2_L(f)$
parameters:	σ_{RF} = stimulus contrast within a unit's responsive freq. range<br>σ_{global} = weighted average stimulus contrast across all frequencies<br>b_{RF}, b_{global} = relative weightings of the above<br>Φ_{RF} = responsive frequency range<br>Φ = set of all frequencies presented<br>$\sigma_L(f)$ = stimulus contrast within freq. band f<br>$n = 1.31$ inherited from #5 above
fitted values:	$b_{RF} = 0.079, b_{global} = 0.033$ (Fig. 7F)
use:	Main text
cross-validated:	Yes. Model fitted on units where the test overlapped their STRF, and successfully predicted the gain exhibited by units where the test only partially overlapped the STRF, or lay completely outside it (Table S4).

Figure S1, related to Figure 1: Relationship between level statistics and sound pressure statistics; graphical representation of gain control model

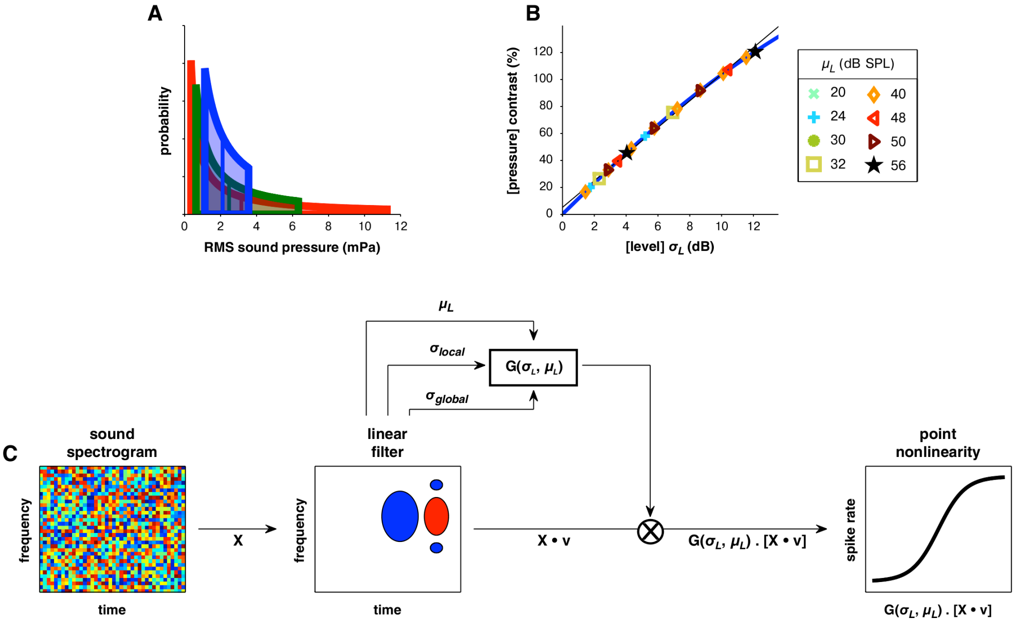

(A) **Distributions of tone sound pressures (mPa) corresponding to the level distributions shown in Figure 1D.** In the (logarithmic) level domain, the blue (low σ_L) distribution is uniform, with a mean μ_L = 40 dB SPL and a width of ± 5 dB; the green (medium σ_L) and red (high σ_L) distributions have the same level mean but level widths of ± 10 and ± 15 dB respectively. In the (linear) sound pressure domain, as shown here, these are no longer uniform distributions, and their means are no longer identical.

(B) The contrast (σ_P/μ_P) of the sound pressure distribution is highly correlated with the standard deviation (σ_L) of the sound level distribution. Colored symbols shown correspond to the range of stimulus statistics presented during this study. Blue thick line shows the analytic solution of the relationship between these variables: $\frac{\sigma_P}{\mu_P} = \sqrt{\xi \coth(\xi) - 1}$, where $\xi = \frac{\ln(10)\sqrt{12}}{40}\sigma_L$ (see Supplemental Experimental Procedures). The black thin line shows a linear fit between the sound pressure contrasts and level standard deviations of the presented distributions. This provides an excellent fit (r^2 = 0.997), indicating that these measures are interchangeable, up to a scale factor and constant offset, i.e. Eqn. (1) in main text.

It is worth noting that this approximation does not necessarily hold under all sets of stimulus statistics, and appears to break down as the kurtosis of the sound level distribution is increased.

Table S3, related to Figure 2: Prediction scores are worse under low contrast stimulation

	Low contrast σ_L = 2.9 dB c = 33%	Intermediate contrast σ_L = 5.8 dB c = 64%	High contrast σ_L = 8.7 dB c = 92%
**% total**			
median signal power	55.1	68.5	71.3
median noise power	44.9	31.5	28.7
**Normalized**			
median signal power	0.47	0.83	1
median noise power	0.99	1.00	1
**explained by STRFs**			
median % signal power	21.3	35.2	34.2
			(n = 115)

As contrast was reduced, fewer units yielded predictive STRFs ($P << 0.001$; Kruskal-Wallis test). In general, units were less reliably driven by low contrast stimuli. Signal power under low contrast stimulation was a median 53% less than that measured under high contrast. Similarly, the intermediate contrast stimulus yielded a signal power that was 17% less than that measured under high contrast. Noise power, however, remained constant across conditions. Thus, units were more reliably driven under high contrast stimulation, and hence their responses were more predictable.

Figure S2, related to Figure 3: Neurons adapt to stimulus contrast by repositioning the maximal sensitivity of their nonlinear output functions

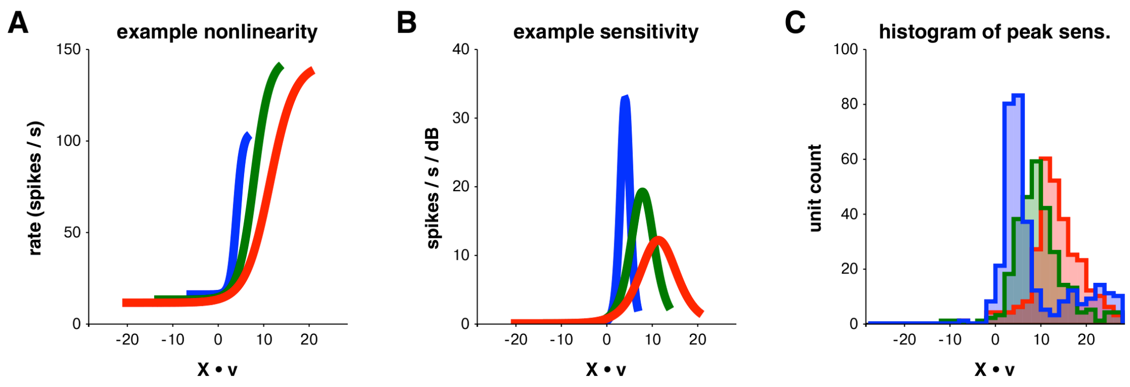

(A-B) The output nonlinearities (A) are not only informative about the overall gain of the units, but also to which region of stimulus space units are most sensitive. The derivative of the nonlinearity with respect to $\mathbf{X} \cdot \mathbf{v}$ (B) relates how a small change in the stimulus impacts the firing rate of the unit. By looking at this derivative as a function of $\mathbf{X} \cdot \mathbf{v}$, we can find the intensity region to which the cell is most sensitive. This is precisely the value of $\mathbf{X} \cdot \mathbf{v}$ where the derivative is greatest.

(C) Although the range of $\mathbf{X} \cdot \mathbf{v}$ values presented in each condition was different – between approximately ±7.4 dB for the low contrast stimulus, ±14.9 dB for the intermediate contrast stimulus, and ±22.3 dB for the high contrast stimulus – we extrapolated from the sigmoids that had been fitted over these limited regions to estimate which stimulus each unit would be most sensitive to under each contrast condition. By this criterion, the maximal slope occurred at a median position of $\mathbf{X} \cdot \mathbf{v}$ = 5.3 / 10.1 / 14.3 dB under low / intermediate / high contrast, respectively. This lies well within the range of $\mathbf{X} \cdot \mathbf{v}$ values presented in the respective conditions, indicating a pronounced shift in the sensitivity profile of neurons in auditory cortex.

Figure S3, related to Figure 4: Controls on LN analysis; contrast gain control for individual cells

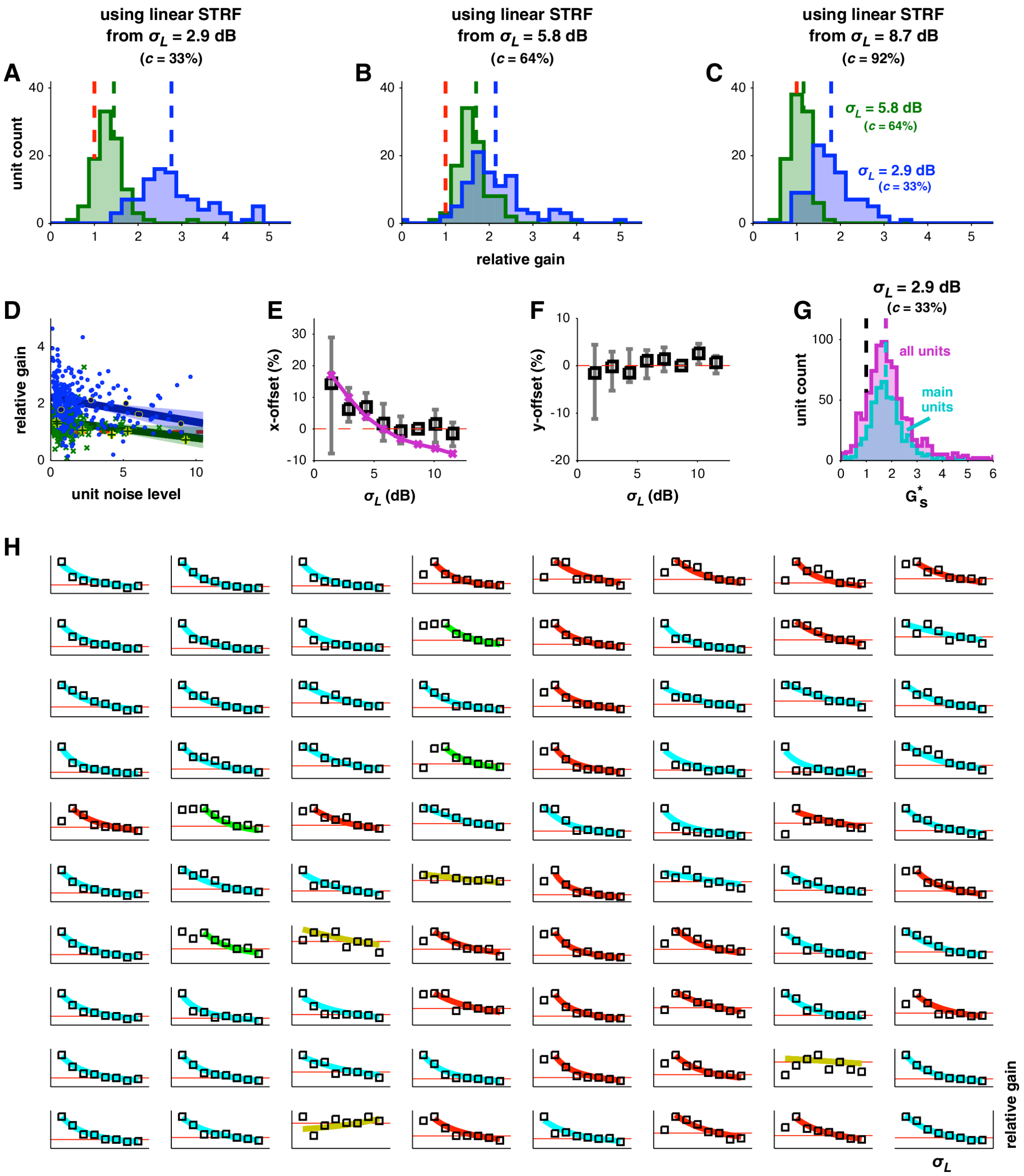

(A-C) **Contrast-dependent gain effects in the LN model are seen regardless of which STRF constitutes the linear component of the model.** The LN model comprises a linear STRF and a point nonlinearity. In the main text, we describe how the data from all conditions were pooled to fit a single linear STRF, and separate nonlinearities were fitted for each contrast condition (Figures 3-4). We validated this procedure by checking that there were no systematic differences between STRF shape in different conditions.

To check that the differences in gain were not a by-product of using a STRF derived from the pooled dataset, we ran the same analysis where the STRF was fitted from only the responses of units to either the low contrast stimuli (A), medium contrast stimuli (B), or high contrast stimuli (C). In each case, the point nonlinearities were then estimated for all three conditions, and the input gain calculated. Panels (A-C) are equivalent to Figure 4A. The same qualitative observations were made in every case, regardless of which STRFs were used: lower stimulus contrast is compensated for by an increase in response gain.

(D) **The gain effect was strongest among units with the most robust, repeatable spike trains: units with low noise levels showed a greater modulation of their gain as a function of contrast.** The measured gain (ordinate) is dependent on the between-trial variability of the data recorded from each unit across multiple presentations of the same stimulus (noise level; abscissa). The more consistent a unit's responses across trials, the greater the observed gain rescaling. Colors as in Figure 2C-E, Fitted lines as in Figure 4D. Red dashed line indicates $G = 1$ across conditions, i.e. no gain modulation. If we extrapolate from the population data to a hypothetical zero-noise neuron (Sahani and Linden, 2003b; Ahrens et al., 2008), we obtain relative gain values of 2.21 ± 0.16 and 1.40 ± 0.06, respectively (median ± 99% confidence interval) for the same two contrast comparisons. This implies that neurons do not completely compensate for stimulus contrast. There was no corresponding dependence of x-offset or y-offset on noise level (data not shown).

(E, F) **Dependence of x-offset and y-offset values on a broad range of stimulus contrasts.** Panels E and F show the relationship between stimulus contrast (σ_L) and x-/y-offset in an equivalent manner to Figure 4E. Magenta line shows the predicted x-offset calculated from the (within-contrast) relationship between gain and x-offset in Figure 4D, and the dependence of gain on σ_L in Figure 4E. The increase in x-offset as contrast is reduced can therefore be explained primarily by the pronounced increase in gain at low contrast. There is no systematic effect of stimulus contrast on y-offset.

(G) **Most units show gain control.** Among 1001 units, 458 yielded predictive LN models in both low and high contrast stimulus conditions, while 554 yielded predictive LN models in both medium and high contrast conditions. For these units, we used the LN model to estimate gain in each condition. For the remaining units, an alternative measure of gain was used (G_S^*; see Supplemental Experimental Procedures, Eqn. (S5)), comparing the gain in low ($\sigma_L = 2.9$ dB, $c = 33\%$) with high ($\sigma_L = 8.7$ dB, $c = 92\%$) contrast conditions. The G_S^* values for the units in the main text (cyan) showed very similar distributions to the G_S^* values for the units for which LN models were not

predictive (purple). The black dashed line at $G_S^* = 1$ denotes no gain; medians of the respective distributions (1.77 and 1.78 respectively) are marked by colored dashed lines.

(H) **Relationship between contrast (abscissa) and gain (ordinate) for 80 units.** Contrast (σ_L) values as in Figure 4E, thin red horizontal line denotes $G = 1$. As contrast was reduced, 46/80 units increased their gain down to the lowest contrast measured, $\sigma_L = 1.4$ dB ($c = 17\%$); 26/80 responded with maximum gain at the second lowest contrast measured ($\sigma_L = 2.8$ dB, $c = 33\%$); four with maximum gain at $\sigma_L = 4.3$ dB ($c = 49\%$); and the remaining four showed no consistent dependence of gain on σ_L. Within the first three of these groups, the input gain could be well described by Eqn. (2) down to their respective maximum-gain contrast values (cyan, red, and green, respectively; yellow fits for the remaining group). This demonstrates that gain normalization is evident on a unit-by-unit basis, although individual units may have limits on how much they can compensate for arbitrarily low stimulus contrasts.

Figure S4, related to Figure 5: Output nonlinearities for two example units show that contrast gain control is weaker at low mean levels; gain effects are identical when expressed in terms of σ_P/μ_P rather than σ_L.

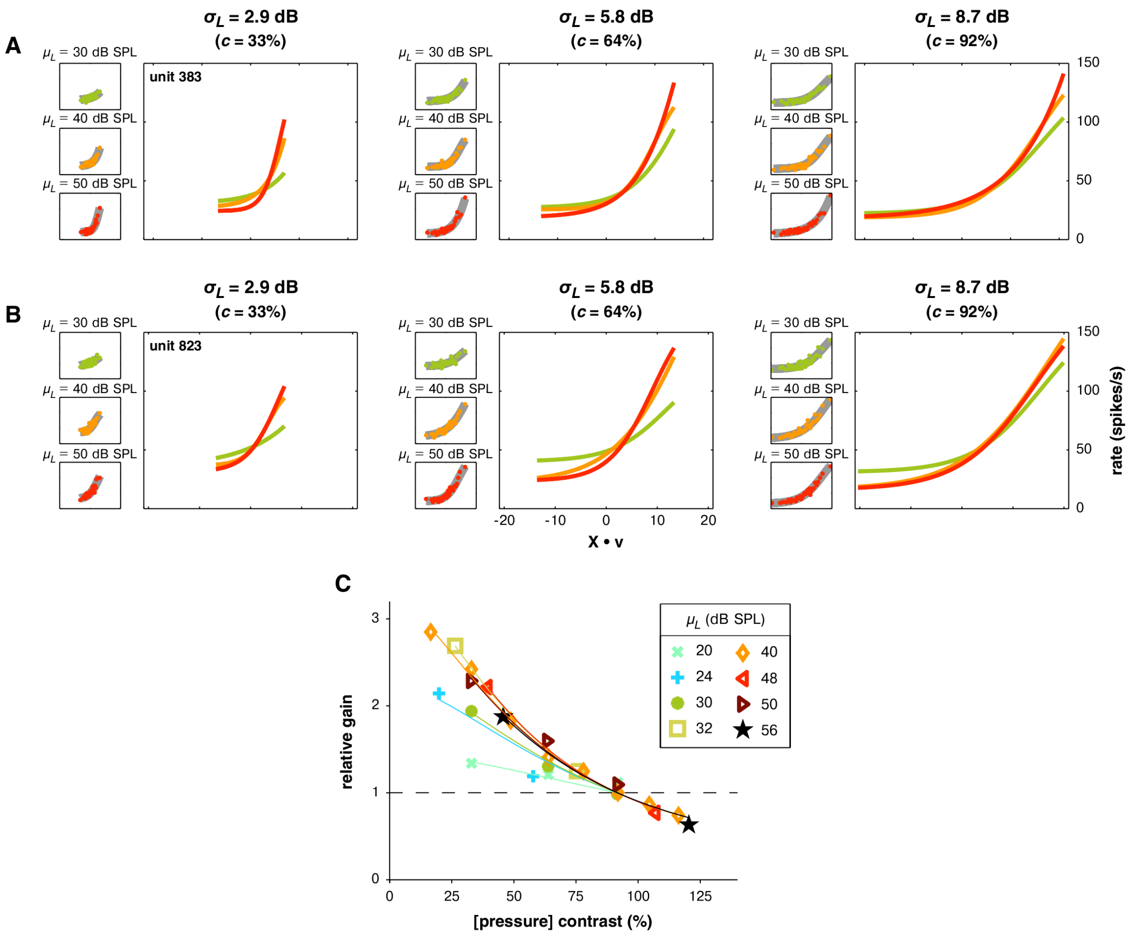

(A) Output nonlinearities for a given unit for three contrast values (left, middle and right blocks), and three stimulus mean levels (small sub-blocks; green μ_L = 30 dB, orange μ_L = 40 dB, red μ_L = 50 dB SPL). Larger panels show all fitted sigmoids for a given contrast but different μ_L values. For this unit, gain rescaling is strong at μ_L = 50 dB and 40 dB, but relatively weak at μ_L = 30 dB.

(B) As in (A), for a second example unit.

(C) As explained in the main text and Figure S1, we used the standard deviation of the distribution of tone levels (σ_L) as a measure of contrast, as this correlates very strongly with the contrast in sound pressure $c = \sigma_P/\mu_P$. For completeness, we provide here a version of Figure 5A where the abscissa is expressed directly in units of $c = \sigma_P/\mu_P$ (%), rather than in units of standard deviation. All analyses and model fitting produced almost identical results when contrast was expressed as a percentage rather than in dB.

Figure S5, related to Figure 6: Excitatory responses to the test sound in low and high contrast context show similar time courses.

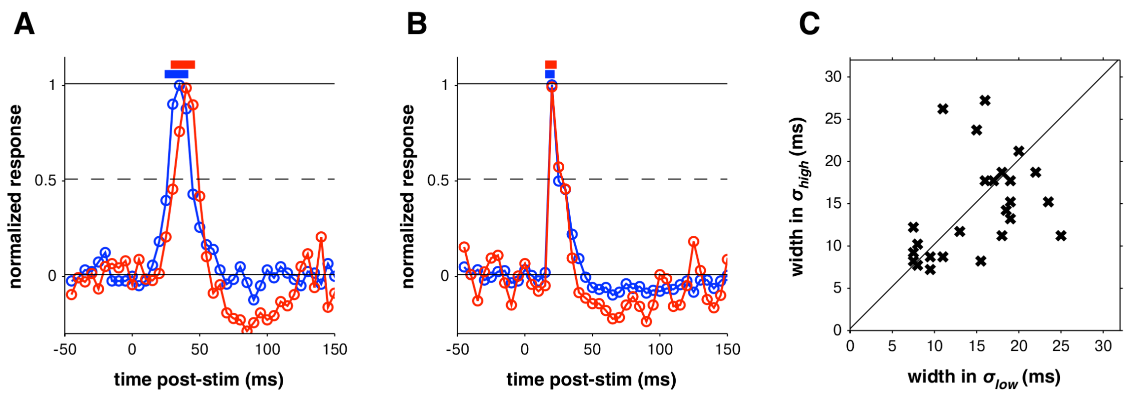

(A) Average response to the test sound for an example unit, as in Figure 6C. Blue circles and blue line show the response when the test sound was embedded in a low contrast context; red circles and red line show response when the test sound was embedded in a high contrast context. Responses have been averaged within each contrast context over all post-switch delays from 150-800 ms. As only the time course of the response is of interest here, rather than the amplitude, responses in both conditions have been normalized to have a background rate of 0 and a peak response of 1. The duration of the excitatory portion of the response is measured as the width at half-maximum (i.e. along the dotted line). These are shown for each condition as the coloured thick bars. For this unit, the durations were 17.5 s in low contrast context, and 18 s in high contrast context.

(B) As in (A), for a second example unit.

(C) Relationship between the duration of the response to the test stimulus in high and low contrast contexts units, measured by the width at half-maximum as in (A,B). To ensure that durations could be accurately measured, only n=25 units where the standard deviation of the background rate was less than a third of the half-maximum firing rate were included. There was no significant difference between these durations (sign-rank, $P > 0.3$).

Table S4, related to Figure 7: Success of test/mask models

Model type	Median %VE; units with the test covering the responsive frequency range	Median %VE: units with the test not covering the responsive frequency range
**single:** $G(\sigma) = \frac{a}{1 + b\,\sigma^n}$		
$\sigma = \sigma_{test}$	60%	-30%
$\sigma = \sigma_{local}$	**62%**	**76%**
$\sigma = \sigma_{mask}$	-16%	62%
$\sigma = \sigma_{remote}$	4%	52%
$\sigma = \sigma_{global}$	7%	72%
**weighted:** $G(\sigma_1, \sigma_2) = \frac{a}{1 + b_1\,\sigma_1^n + b_2\,\sigma_2^n}$		
$\sigma_1 = \sigma_{test}$, $\sigma_2 = \sigma_{mask}$	73%	11%
$\sigma_1 = \sigma_{local}$, $\sigma_2 = \sigma_{remote}$	65%	83%
$\sigma_1 = \sigma_{local}$, $\sigma_2 = \sigma_{global}$	**71%**	**83%**

For the test/mask analysis, we fit two classes of models to a training dataset. This dataset consisted of the relative gain values for the 9 test/mask stimulus conditions, averaged (median) across 24 units where the test completely covered the responsive frequency range. The models included contributions to the gain from a combination of either one or two of:

- the test contrast, σ_{test}
- the mask contrast, σ_{mask}
- a measure of local contrast, defined via the average level variance within the frequency bands constituting a unit's responsive frequency range (Φ_{RF}):

$$\sigma_{local}^2 = \frac{1}{|\Phi_{RF}|} \sum_{f \in \Phi_{RF}} \sigma_L^2(f)$$

- a measure of non-local ("remote") contrast, defined via the average level variance within the frequency bands outside the unit's responsive frequency range ($\Phi_{outside}$):

$$\sigma_{remote}^2 = \frac{1}{|\Phi_{outside}|} \sum_{f \in \Phi_{outside}} \sigma_L^2(f)$$

- a measure of the global contrast, defined via the average level variance across all frequency bands (Φ):

$$\sigma_{global}^2 = \frac{1}{|\Phi|} \sum_{f \in \Phi} \sigma_L^2(f)$$

where $\sigma_L(f)$ is the contrast in frequency band *f*. The sets Φ_{RF} and $\Phi_{outside}$ were determined by dividing the 34 frequency bands of Φ into those for which $\mathbf{w}_f$ had a significantly non-zero component via the bootstrap procedure documented in Experimental Procedures. The choice of the exponent of $\sigma_L(f)$ in the weighted sums was arbitrary; our range of conditions was insufficient to constrain this. Similar results were obtained when these weighted sums were of the form $\sigma_i = \frac{1}{|\Phi_i|} \sum_{f \in \Phi_i} \sigma_L(f)$. For fitting the model to the population median data, it was assumed that $\sigma_{local} = \sigma_{test}$ and $\sigma_{remote} = \sigma_{mask}$. The value of $n = 1.31$ was fixed.

Each of the fitted models documented in the table were tested via a validation and a prediction. Firstly, the model, whose parameters were fit on the population median, was used to calculate the expected relative input gain of the 24 individual units where the test completely covered Φ_{RF}. This used the unit-by-unit measurement of the five contrast measures as listed above. The median percentage of variance explained across these units (%VE) is reported in the middle column of the table. Secondly, the model was used to predict the relative input gain for 42 individual units with Φ_{RF} lying entirely outside the test (rightmost column of the table). Again, this used the unit-by-unit measurement of the five contrast measures as listed above.

Two major categories of models were evaluated. In the first category, models could depend on only one measure of stimulus contrast. Here, a dependence on σ_{local} provided the best validation and prediction scores (bold text in highlighted row), indicating that neural gain is most powerfully modulated by stimulus variance within the frequency bands closest to its BF.

In the second category, models could depend on both the stimulus contrast in local frequency bands, and in remote bands (or all bands), via a weighted sum in the denominator of the gain equation. Better validation and prediction scores were provided by including σ_{global} as well as σ_{local}, compared with the σ_{local} model only. This demonstrates that neuronal gain is not just determined by the statistics within the responsive frequency range of a neuron. Notably, the weighting in the denominator on σ_{local} is 2.4x greater than for σ_{global}, showing that local effects remain stronger than global effects.

Supplemental Experimental Procedures

Spike sorting

Off-line spike sorting was performed using spikemonger, an in-house software package for Matlab (Mathworks Inc, Natick MA). Candidate spikes were identified as voltage-threshold crossing events. An automated expectation maximization algorithm sorted spikes by shape across up to five channels. Multi-unit clusters were chosen for further analysis; 42% (427/1020) of these were deemed to be single units based on spike shape and the presence of a refractory period in the autocorrelation histogram. We only included units that displayed acoustically-responsive activity.

Analytic relationship between sound pressure statistics and level statistics

The distributions of tone levels in this study were defined as uniform distributions in the level domain (Figure 1D), i.e.:

$$P(L) = \begin{cases} 1/(2w) & \mu - w \le L < \mu + w \\ 0 & \text{otherwise} \end{cases} \qquad \text{(S1)}$$

where μ is mean level, and w is the half-width of the level distribution. In the pressure domain, these distributions become:

$$P(L) = \begin{cases} 1/(2\,\xi\, p) & \nu e^{-\xi} \le p < \nu e^{\xi} \\ 0 & \text{otherwise} \end{cases} \qquad \text{(S2)}$$

with the transformed variables $\xi = w\ \ln(10)/20$ and $\nu = p_0\,.\,10^{\mu/20}$, and the standard reference RMS sound pressure, p_0 = 20 μPa. Computing expectations gives $E[p] = \frac{\nu}{\xi}\sinh(\xi)$ and $E[p^2] = \frac{\nu^2}{2\xi}\sinh(2\xi)$. The square of the contrast can then be computed by $C^2 \equiv \left(\frac{\sigma_p}{\mu_p}\right)^2 = \frac{E[p^2]}{E[p]^2} - 1$, giving $C = \sqrt{\xi\,.\coth(\xi) - 1}$.

Statistics on STRFs

Statistics on the STRF were calculated as follows. The best frequency (BF) was calculated as the center of mass (CoM) of the (element-wise) squared frequency kernel, $\mathbf{w}_f^2$. Given that a finite range of frequencies was used, noise in the estimates of $\mathbf{w}_f^2$ exerted a bias in the CoM values towards the center of this range. To compensate for this bias, we calculated the CoM on a circular basis. We manually checked that there were no wrapping artifacts when the true BF was at either the lower or upper extreme of the frequency range. Bandwidth was estimated as the (circular) standard deviation of $\mathbf{w}_f^2$. Circular statistics were performed using CircStat in Matlab (Berens, 2009). As we found that noise in the STRF had a considerable impact on the estimate of bandwidth, we calculated a second bandwidth measure after excluding all coefficients of $\mathbf{w}_f^2$ that were not significantly non-zero. This was established via a bootstrap procedure, described below. We confirmed that all results held when BFs and bandwidths were estimated on non-circular (i.e. standard) bases.

Bootstrapped estimates of non-zero components of the STRFs

To determine what proportion of each STRF lay within the test, we first constructed confidence intervals for the components of $\mathbf{w}_f$ via bootstrapping. We drew (with replacement) 1000 subsamples of 90% of the stimulus/response data from the pooled dataset, and estimated a STRF from each. Those components of $\mathbf{w}_f$ not significantly nonzero across the ensemble (via studentization, P < 0.01) were set to zero. Equation (4) as given in the main text was calculated using only the non-zero components of $\mathbf{w}_f$.

Fitting of curve transformations

We fit Eqn. (6) in the Experimental Procedures by minimizing a quadratic loss function. This measured the squared difference between the target curve, $F_{\sigma_L}\left[\mathbf{X}\cdot\mathbf{v}\right]$, and the transformed reference curve $F_{\sigma_0}\left[g\ .\ \left(\mathbf{X}\cdot\mathbf{v}\right)+\Delta x\right]+\Delta y$, over the domain of the target curve only. We used gradient descent to find the optimal parameters of the gain, g, the x-offset, Δx, and the y-offset, Δy, using initial conditions of $g_0 = 1, \Delta x_0 = 0, \Delta y_0 = 0$. Gradient descent was repeated a further ten times from random initial conditions (with $g_0 \sim \mathrm{Exp}(1)$, and $\Delta x_0, \Delta y_0 \sim N(0,10^2)$), and the best of all these fits chosen. Results were cross-checked by minimizing the mean squared error between the transformed curve and the original data by which the original sigmoid was fit.

Though curve transformations could, in principle, include both a scaling of curves along both the horizontal and vertical axes, the extent of vertical scaling could not be accurately estimated alongside horizontal scaling as none of the units measured operated near their saturation point. Nevertheless, we considered whether changes in the nonlinearities could be explained by vertical scaling (together with x- and y-offsets), rather than by horizontal scaling. This amounted to fitting the equation:

$$F_{\sigma_L}\left[\mathbf{X}\cdot\mathbf{v}\right] = g \,.\, F_{\sigma_0}\left[(\mathbf{X}\cdot\mathbf{v})+\Delta x\right] + \Delta y \qquad \text{(S3)}$$

in the same manner as Eqn (5) in the main text (see details above). This provided considerably poorer fits than Eqn (5) (data not shown).

Gain effects on units without predictive LN models

In the main text, we measure gain changes by measuring the horizontal scaling of output nonlinearities between different conditions. In order to be able to perform this analysis for a unit, we need a LN model that predicts the unit's responses in all conditions. For many units in our sample, we were unable to estimate such a model, and so we could not measure gain changes in this way. To assess gain changes across the whole sample, we performed the following alternative analysis.

Consider a sample of noiseless neurons, with linear stimulus-response relationships. If the neurons do not have contrast gain control, then we should expect the standard deviation of neural responses, $\sigma^{(R)}$, to be directly proportional to the standard deviation of the stimulus, $\sigma^{(S)}$. On the other hand, if neurons have complete contrast gain control, then any reduction in stimulus contrast should be compensated for by an increase in gain, so that $\sigma^{(R)}$ is unaffected by contrast. Thus, we can measure the strength of gain control by comparing $\sigma^{(R)}$ for two stimuli of high and low contrast. By measuring the ratio of response standard deviation in the two conditions ($\sigma_{lo}^{(R)}/\sigma_{hi}^{(R)}$), and comparing it to the corresponding ratio of stimulus standard deviations ($\sigma_{lo}^{(S)}/\sigma_{hi}^{(S)}$), we derive a measure of gain, G^*:

$$G^* = \frac{\sigma_{lo}^{(R)}/\sigma_{hi}^{(R)}}{\sigma_{lo}^{(S)}/\sigma_{hi}^{(S)}} \qquad \text{(S4)}$$

In principle, we can then measure G^* for $\sigma_{lo}^{(S)} = 2.9\,\text{dB}$ and $\sigma_{hi}^{(S)} = 8.7\,\text{dB}$ respectively. If $G^* = 1$, this indicates that there is no gain control ($\sigma^{(R)}$ is proportional to

$\sigma^{(S)}$). If $G^* = 3$, this indicates complete compensation for changes in stimulus standard deviation.

Real neurons are not noiseless, and so $\sigma^{(R)}$ is not a good estimate of the stimulus-driven variability (signal power) in the responses. However, Sahani & Linden (2003b) present a simple method for estimating the signal power and the noise power of a set of neural responses. We used this technique to refine the equation above, by replacing $\sigma^{(R)}$ (as derived from the average response) with $s = \sqrt{\text{signal power}}$:

$$G_S^* = \frac{s_{lo}/s_{hi}}{\sigma_{lo}^{(S)}/\sigma_{hi}^{(S)}} \qquad \text{(S5)}$$

To verify that this indeed produced a useful measure of gain, we compared G_S^* with relative gain (as measured through curve transformations in the main text) for the 458 units where the LN models were predictive in the low and high contrast conditions. Amongst these units, there was a moderate correlation of $r = 0.44$ between these two measures. This suggests that G_S^* can provide a fair picture of gain control amongst the remaining units.

Figure S3G shows the resulting values of G_S^* for the 458 units in the main text (cyan), and for the whole sample (purple). The medians of both distributions (1.77 and 1.78 respectively) are significantly greater than 1, indicating that neurons generally show some gain control. Moreover, only 98/1001 neurons have $G_S^* \leq 1$, indicating that gain control is exhibited by the vast majority of units in our sample.

Test sound reliability

To ensure that reliable estimates of time constants were obtained, we limited our analysis of adaptation time course to units that satisfied two criteria. Firstly, it was necessary that the mean peak response was different in the high and low contrast context conditions. Any fitting of exponentials of the form $r(t) = a + b\,.\exp(-t/\tau)$ to units where there was no difference between peak responses in high and low conditions would only be able to capture noise. Secondly, we calculated the noise ratio (NR) (defined in Sahani and Linden, 2003b; see Experimental Procedures) of the set of peak responses to the test sound, across all conditions for that unit. As for the PSTHs, NR=0 indicates that the peak responses were identical for each repeated stimulus presentation, while higher NR indicates that responses were less reliable. As for the STRFs, we restricted our analysis to units whose NR for peak responses was < 10.
